# *SYT1-*associated neurodevelopmental disorder: a case series

**DOI:** 10.1093/brain/awy209

**Published:** 2018-08-13

**Authors:** Kate Baker, Sarah L Gordon, Holly Melland, Fabian Bumbak, Daniel J Scott, Tess J Jiang, David Owen, Bradley J Turner, Stewart G Boyd, Mari Rossi, Mohammed Al-Raqad, Orly Elpeleg, Dawn Peck, Grazia M S Mancini, Martina Wilke, Marcella Zollino, Giuseppe Marangi, Heike Weigand, Ingo Borggraefe, Tobias Haack, Zornitza Stark, Simon Sadedin, Broad Center for Mendelian Genomics, Tiong Yang Tan, Yunyun Jiang, Richard A Gibbs, Sara Ellingwood, Michelle Amaral, Whitley Kelley, Manju A Kurian, Michael A Cousin, F Lucy Raymond

**Affiliations:** 1 Department of Medical Genetics, Cambridge Institute for Medical Research, University of Cambridge, Cambridge Biomedical Campus, Wellcome Trust / MRC Building, Hills Road, Cambridge, UK; 2 MRC Cognition and Brain Sciences Unit, 15 Chaucer Road, Cambridge, UK; 3 The Florey Institute of Neuroscience and Mental Health, University of Melbourne, 30 Royal Parade, Parkville, VIC, Australia; 4 Department of Biochemistry and Molecular Biology, University of Melbourne, 30 Royal Parade, Parkville, VIC, Australia; 5 Department of Clinical Biochemistry, Cambridge Institute for Medical Research, Hills Road, Cambridge, UK; 6 Developmental Neurosciences, UCL Great Ormond Street Institute of Child Health, 30 Guilford Street, London, UK; 7 Ambry Genetics, 15 Argonaut, Aliso Viejo, CA, USA; 8 Department of Clinical Genetics, Queen Rania Al-Abdullah Children Hospital, King Hussein Medical Centre, Royal Medical Services, Amman, Jordan; 9 Monique and Jacques Roboh Department of Genetic Research, Hadassah, Hebrew University Medical Center, Jerusalem, Israel; 10 University of Missouri Health Care, Columbia, MO, USA; 11 Department of Clinical Genetics, Erasmus Medical Center, 3015 CN Rotterdam, The Netherlands; 12 Institute of Genomic Medicine, Catholic University, A. Gemelli Foundation, Roma, Italy; 13 Department of Pediatric Neurology, Developmental Medicine and Social Pediatrics, Dr. von Hauner’s Children’s Hospital, University of Munich, Munich, Germany; 14 Institute of Human Genetics, Technische Universität München, Munich, Germany; 15 Institute of Medical Genetics and Applied Genomics, University of Tuebingen, Tuebingen, Germany; 16 Victorian Clinical Genetics Services, Murdoch Children’s Research Institute, Flemington Road, Parkville VIC, Australia; 17 Program in Medical and Population Genetics, Broad Institute of MIT and Harvard, Cambridge, Massachusetts, USA; 18 Broad Center for Mendelian Genomics, Cambridge, Massachusetts, USA; 19 Human Genome Sequencing Center, Baylor College of Medicine, Texas, USA; 20 Maine Medical Partners Pediatric Specialty Care, Congress St, Portland ME, USA; 21 HudsonAlpha Institute for Biotechnology, 601 Genome Way NW, Huntsville, AL, USA; 22 Centre for Discovery Brain Sciences, Hugh Robson Building, George Square, University of Edinburgh, Edinburgh, UK

**Keywords:** *SYT1*, synaptotagmin 1, movement disorder, intellectual disability, synaptic vesicle

## Abstract

Synaptotagmin 1 (SYT1) is a critical mediator of fast, synchronous, calcium-dependent neurotransmitter release and also modulates synaptic vesicle endocytosis. This paper describes 11 patients with *de novo* heterozygous missense mutations in *SYT1.* All mutations alter highly conserved residues, and cluster in two regions of the SYT1 C2B domain at positions Met303 (M303K), Asp304 (D304G), Asp366 (D366E), Ile368 (I368T) and Asn371 (N371K). Phenotypic features include infantile hypotonia, congenital ophthalmic abnormalities, childhood-onset hyperkinetic movement disorders, motor stereotypies, and developmental delay varying in severity from moderate to profound. Behavioural characteristics include sleep disturbance and episodic agitation. Absence of epileptic seizures and normal orbitofrontal head circumference are important negative features. Structural MRI is unremarkable but EEG disturbance is universal, characterized by intermittent low frequency high amplitude oscillations. The functional impact of these five *de novo SYT1* mutations has been assessed by expressing rat SYT1 protein containing the equivalent human variants in wild-type mouse primary hippocampal cultures. All mutant forms of SYT1 were expressed at levels approximately equal to endogenous wild-type protein, and correctly localized to nerve terminals at rest, except for SYT1^M303K^, which was expressed at a lower level and failed to localize at nerve terminals. Following stimulation, SYT1^I368T^ and SYT1^N371K^ relocalized to nerve terminals at least as efficiently as wild-type SYT1. However, SYT1^D304G^ and SYT1^D366E^ failed to relocalize to nerve terminals following stimulation, indicative of impairments in endocytic retrieval and trafficking of SYT1. In addition, the presence of SYT1 variants at nerve terminals induced a slowing of exocytic rate following sustained action potential stimulation. The extent of disturbance to synaptic vesicle kinetics is mirrored by the severity of the affected individuals’ phenotypes, suggesting that the efficiency of SYT1-mediated neurotransmitter release is critical to cognitive development. In summary, *de novo* dominant *SYT1* missense mutations are associated with a recognizable neurodevelopmental syndrome, and further cases can now be diagnosed based on clinical features, electrophysiological signature and mutation characteristics. Variation in phenotype severity may reflect mutation-specific impact on the diverse physiological functions of SYT1.

## Teaser

Baker, Gordon *et al*. present the first international case series describing the neurodevelopmental disorder associated with Synaptotagmin 1 (*SYT1*) *de novo* missense mutations. Key features include movement abnormalities, severe intellectual disability, and hallmark EEG alterations. Expression of patients’ *SYT1* mutations in mouse neurons disturbs presynaptic vesicle dynamics in a mutation-specific manner.

## Introduction

Healthy brain function relies on tight regulation of the probability and timing of neurotransmitter release (Waites and Garner, 2011; Jahn and Fasshauer, 2012). A fundamental step in this pathway is the calcium-dependent triggering of fusion between synaptic vesicle and plasma membranes to enable coordinated fast neurotransmitter release. The synaptotagmins are a family of integral synaptic vesicle proteins required for the synchronous coupling of activity-dependent calcium influx to synaptic vesicle fusion at central synapses. Synaptotagmin 1 (SYT1) is the primary cerebral isoform, expressed throughout the neocortex and subcortical structures in postnatal life (http://www.braineac.org/). SYT1 triggers synaptic vesicle fusion by binding calcium via highly conserved cytoplasmic C2A and C2B domains, followed by penetration of the plasma membrane bilayer by a series of hydrophobic residues within these domains (Sudhof, 2013). In addition, SYT1 plays a modulatory role in endocytosis (Poskanzer *et al.*, 2003; Yao *et al.*, 2011), and has recently been implicated in the calcium-sensitive trafficking of postsynaptic AMPA receptors to facilitate long term potentiation (Wu *et al.*, 2017). SYT1 therefore influences multiple aspects of synaptic physiology necessary for neurotransmission and synaptic plasticity.

We previously described the first case of a human disorder associated with a rare variant in *SYT1* (Baker *et al.*, 2015)*.* The individual harbouring a *de novo* mutation (I368T) presented with an early onset mixed hyperkinetic movement disorder, severe motor delay, and profound cognitive impairment. Structural MRI was normal, but EEG showed extensive neurophysiological disturbances. Expression of rat SYT1 containing the equivalent human mutation in wild-type mouse primary hippocampal cultures altered the kinetics of exocytosis and endocytosis, in agreement with the role for I368 in calcium-dependent membrane penetration (Paddock *et al.*, 2011). A second *de novo* missense variant in *SYT1* (M303K) was identified within a series of patients with dysmorphology and developmental delay (Cafiero *et al.*, 2015). Neurological symptoms were not reported for this second case, motor milestones were less severely delayed, and cognitive impairment was also less severe. As in the first case, EEG abnormalities were reported despite no overt seizures.

Defining the syndrome associated with mutations in *SYT1* requires validation by the identification of further individuals with similar mutation characteristics and phenotypic features. To this end, we now report medical, neurological and developmental phenotypes for the two previously reported patients and nine new patients with *de novo SYT1* mutations. This case series provides sufficient evidence that rare missense mutations in *SYT1* are associated with a distinctive neurodevelopmental phenotype and EEG abnormality. The presence of recurrent mutations clustered around the calcium-binding pocket of the C2B domain suggests potential mechanisms of disease. Functional assessment of the five mutations further supports pathogenicity, and points toward genotype-specific synaptic pathophysiology.

## Materials and methods

### Patient identification and consent

Identification of patients with *de novo SYT1* mutations began after reporting the first case (Baker *et al.*, 2015). The cohort reported here emerged from direct contact from clinicians and genomics research groups with similar cases recognized from reporting the first case. Patients were selected by clinicians in 10 different centres for diagnostic investigation via exome sequencing or genome sequencing, on either a clinical or research basis, in view of unexplained neurodevelopmental disorders.

Consent for genetic testing was obtained via approved procedures at each local contributing centre. After genetic diagnosis, written consent to collate and report clinical data was obtained from parents or guardians under Cambridge Central Research Ethics Committee approval (IRAS 83633, REC ref: 11/0330/EE), plus specific additional consent for publication of patients’ photographs or videos.

### Sequencing methods

The methodology of variant identification for Patient 1 has been previously published, using trio analysis of a customized whole exome bait (Agilent Technologies) designed for the UK10K Project (Baker *et al.*, 2015). Variants for Patients 2 and 5 were identified via trio exome analysis using Agilent Sureselect Exome V4, with average read depth coverage of 50–100×. Patients 3, 4 and 10 were identified by GeneDx sequence provider (Gaithersburg, USA) using trio analysis with Agilent Clinical Research Exome as bait, with mean exome coverage of 165×, 84× and 106×, respectively. The methodology for Patient 6 was previously reported, using a SureSelect Mbp All exon kit 2.0 (Agilent Technologies) (Cafiero *et al.*, 2015). Patient 7’s variant was identified via trio exome analysis using Agilent Sureselect Exome V5. Patient 8’s variant was identified via trio exome analysis using Illumina exome capture (38 Mb target) at the Broad Institute, Cambridge USA. Patient 9’s variant was identified by trio exome sequencing at the Human Genome Sequencing Center at Baylor College of Medicine using the Nimblegen SeqCap EZ HGSC VCRome Kit. Patient 11’s variant was identified by whole genome sequence analysis at HudsonAlpha Institute of Biotechnology, using non-amplified genomic DNA in the Illumina HiSeq X Ten sequencing system, with 150 bp paired-end reads with a minimum coverage of 20× per base for 80% of bases.

In all cases the *de novo SYT1* variant was identified using standard variant calling and rare variant annotation methods. All *de novo SYT1* variants were confirmed by either Sanger sequencing or repeat exome analysis using an independent pull-down method (Patient 8). In all cases this was the only likely pathogenic rare *de novo* variant reported. There were no likely pathogenic X-linked variants in male patients.

### Molecular modelling and molecular dynamics simulations

To investigate the potential impact of mutations on the structure of the SYT1 C2B domain, >1 µs molecular dynamics simulations were performed on C2B models derived from a Ca^2+^-bound solution nuclear magnetic resonance (NMR) structure (PDB 1k5w; note that amino acid numbering used follows human sequence for simplicity) generated using Molsoft ICM Pro (for full details see Supplementary material). The root-mean-square deviations (RMSD) of the backbone atoms of each SYT1 C2B domain variant, compared to the starting structures, were plotted over the complete trajectories of the simulations (Supplementary Fig. 1A). This measures the average variations in distances between the backbone atoms of each protein over time, providing a readout of the change in protein structure over time. The Ca^2+^-binding ability of the C2B domains was analysed by tracking the distances between the bound Ca^2+^ ions and the gamma carbon of Asp363 (equivalent to human Asp364) throughout the trajectories (Supplementary Fig. 1B and C).

### Clinical phenotyping

Historic and contemporary neurological and neurodevelopmental records were reviewed for all patients. A list of Human Phenotype Ontology terms found to be associated with *SYT1* mutation is provided in Supplementary Table 1 (Kohler *et al.*, 2017). Where possible, video recordings of patients were supplied for neurological review. All EEG recordings and reports were reviewed by a paediatric neurophysiologist.

### Functional studies

Site-directed mutagenesis was used to introduce the human mutations into the homologous position in rat SYT1 (human/rat: M303/302>K, D304/303>G, D366/365>E, N371/370>K, amino acid numbering used henceforth follows human sequence), which was fused to a pH-sensitive EGFP (pHluorin) at its lumenal N-terminus. Mutagenesis was performed using QuikChange II Site-directed Mutagenesis kit (Agilent Technologies); mutagenic primers are listed in Supplementary Table 2, and mutations were confirmed by sequencing. SYT1^I368T^–pHluorin was made as previously described (Baker *et al.*, 2015).

Dissociated primary hippocampal-enriched neuronal cultures were prepared from embryonic Day 16.5–18.5 C57BL/6 J mouse embryos as described (Baker *et al.*, 2015; for full details see Supplementary material). Cells were transfected after 7–8 days in culture and were used for fixation or live cell imaging assays after 13–16 days in culture.

For SYT1 expression and localization assays, neurons were first washed with saline buffer and then either fixed immediately (basal), exposed to 50 mM KCl buffer for 30 s and then fixed immediately (KCl depolarization), or exposed to 50 mM KCl buffer for 30 s and then allowed to recover in saline buffer for 2.5 min before being fixed (recover) (all performed at 37°C) and immunolabelled (further details are available in the Supplementary material).

Live fluorescence imaging assays were performed using SYT1-pHluorin. Cultures were stimulated with a train of 1200 action potentials at 10 Hz in saline buffer or high Ca^2+^ buffer (4 mM CaCl_2_ in place of 2 mM CaCl_2_), supplemented with the V-type ATPase inhibitor 1 μM bafilomycin A1. Further information regarding acquisition conditions and analysis are detailed in the Supplementary material.

All statistical analyses were performed using Microsoft Excel and GraphPad Prism software. One-way, two-way or repeated measures ANOVA with Dunnett’s multiple comparison test was used for data comparing mutants to wild-type protein, or with Tukey’s multiple comparison test to compare changes in SYT1 localization and for experiments performed at different Ca^2+^ concentrations. *P < *0.05 was considered significant.

### Data availability

The primary genetic data that support the findings of this study are openly available for Patient 1 in the European Genome-phenome Archive (https://www.ebi.ac.uk/ega/home) within study accession number EGAS00001000128, dataset accession EGAD00001000416. Data for Patients 8, 9 and 11 are available in dbGaP (https://www.ncbi.nlm.nih.gov/gap) within project numbers phs001272, phs000711.v5.p1 and phs001089, respectively. Data for the remaining participants are held within clinical diagnostic services and are not publicly available. All reported *SYT1* variants have been deposited in ClinVar (https://www.ncbi.nlm.nih.gov/clinvar/).

## Results

### *SYT1* variant evaluation

**Figure 1:**
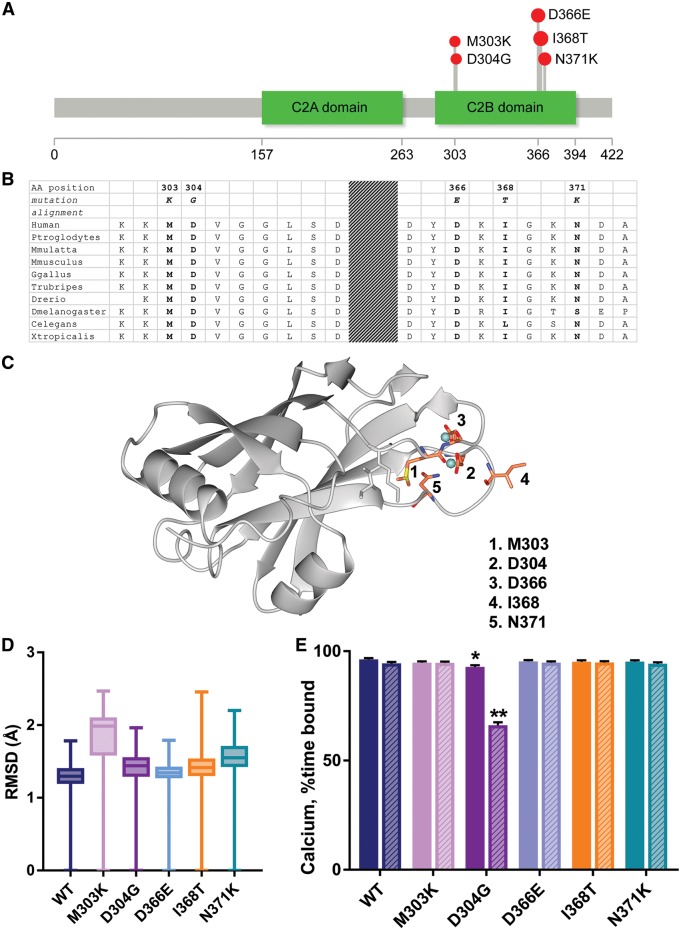
***SYT1 de novo* mutations cluster in the C2B domain.** (**A**) 2D cartoon of SYT1 domain structure depicting position of patients’ mutations (Jay and Brouwer, 2016). (**B**) Evolutionary conservation at mutation sites. (**C**) Structure of the C2B domain of SYT1 in ribbon representation coloured grey (McNicholas *et al.*, 2011). Mutated residues are shown in ball and stick representation coloured in atom colour (carbons orange) and Ca^2+^ ions in cyan. The side chains of Ile374 and Lys301, which pack against Met303, are shown in grey ball and stick representation. (**D** and **E**) Simulations (1.3 µs) were performed on C2B models derived from the calcium-bound soluble NMR structure (PDB 1k5w). Models of the mutant C2B domains were generated using Molsoft ICM Pro. (**D**) The average RMSD of the backbone atoms of each protein, compared to the starting structures, across all frames in the simulations. Data are mean ± standard error of the mean (SEM). (**E**) The Ca^2+^-binding ability of the C2B domains was analysed by tracking the distances between the bound calcium ions and the gamma carbon of Asp363 (equivalent to human Asp364) throughout the trajectories. Data are percentage occupancies ± SEM at the calcium 1 (filled) and calcium 2 (striped) sites for each protein over the simulation time; calcium ions were considered bound if the distances between the ions and the gamma carbon of Asp363 were <6 Å. *P < 0.01, **P < 0.0001 versus wild-type, two-way ANOVA with Dunnett’s multiple comparisons tests.

Seven different rare *de novo* non-synonymous variants were identified in 11 patients (Table 1), resulting in five different amino acid substitutions (M303K *n = *1, D304G *n = *1, D366E *n = *3, I368T *n = *4, N371K *n = *2). All variants are absent from GnomAD database (version 2.0, accessed 3 September 2017) (http://gnomad.broadinstitute.org) (Lek *et al.*, 2016). At position 371 a common synonymous variant is documented, but no non-synonymous changes have been observed to date amongst healthy controls. A single loss of function variant at position 303 is reported. The protein overall is constrained for missense and loss of function variation (http://exac.broadinstitute.org/). Mutations are clustered in one of two regions of the protein (Fig. 1A). All mutations occur at residues highly conserved throughout evolution and in addition are located within highly conserved blocks of amino acids within the protein (Fig. 1B).

**Table 1:** Clinical characteristics of patients with *de novo SYT1* mutations

Patient number^a^	Patient 6	Patient 3	Patient 10	Patient 2	Patient 11	Patient 1	Patient 4	Patient 9	Patient 8	Patient 7	Patient 5
SYT1^b^ mutation	M303K c.908T>A	D304G c.911A>G	I368T c. 1103T>C	I368T c. 1103T>C	I368T c.1103T>C	I368T c. 1103T>C	D366E c.1098C>A	D366E c.1098C>A	D366E c.1098C>G	N371K c.1113C>G	N371K c.1113C>G
Age at last evaluation, years	7	21	3	4	6	14	3	3	9	3	4
Congenital abnormalities	Joint laxity	Progressive contractures, scoliosis	Nil	Nil	Dermoid cyst	Bilateral talipes	Laryngomalacia, atrial septal defect	Nil	Unilateral 2/3 toe syndactyly, lumbar lordosis, bilateral hindfoot valgus deformities	Dermoid cyst	Nil
Weight, percentile	25th	0.4th	10th	Not available	<3rd	50th	25th	3rd	75th	Not available	Not available
Height, percentile	75th	Not available	10th	Not available	10th	50th	50th	Not available	25th	Not available	Not available
Orbitofrontal circumference, percentile	10th	50th	10th	Not available	5th	25th	75th	75th	50th	Normal range	15th
Medical problems	Nil	Gastro-oesophageal reflux	Gastro-oesophageal reflux	Nil	Gastro-oesophageal reflux, feeding difficulties	Hyperventilation- induced cyanotic episodes	Sleep apnoea	Constipation	Central apnoea	Feeding difficulties	Gastro-oesophageal reflux. Sleep apnoea
Ophthalmic features	Esotropia	Strabismus Hypermetropia	Nystagmus	Bilateral hypermetropia	Nystagmus	Esotropia (repaired)	Esotropia (repaired)	Nystagmus, strabismus	Esotropia	Nystagmus	Nystagmus
Seizures	No	No	No	No	No	No	No	No	No	No	No
Infant hypotonia	Yes	Yes	Yes	Yes	Yes	Yes	Yes	Yes	Yes	Yes	Yes
Movement disorder	Ataxia	Stereotypies	Repetitive leg kicking, foot posturing, stereotypies	Dystonia, chorea-athetosis	Hyperkinesis, stereotypies	Dystonia, chorea-athetosis, stereotypies	-	-	-	Dystonia, dyskinetic cerebral palsy	Trunk and limb dystonia, chorea
Age sat, months	Mild delay	36	20	20	> 12	48	13	24	20	36	-
Age crawled, years	Mild delay	7	-	-	> 2	-	1.3	-	-	-	-
Age walked, years	2	-	-	-	-	10	2.5	-	4.5	-	-
Speech, words used	50	0	0	0	0	0	3	0	0	0	0
Behaviour	Angry outbursts, impatience and impulsivity	Head-banging, object mouthing, chest-beating	Thumb biting, head butting	Finger-chewing	Hand-chewing, object mouthing	Hand-biting, chest-beating	Object mouthing, head-banging	Bites and scratches self when frustrated	Hand biting, screaming, obsessions and repetition	Screaming episodes, hand-chewing	Teeth-grinding, screaming episodes

^a^Patient number reflects order of ascertainment.

^b^Human SYT1 Ref seq: NM_005639; UniProtKB - P21579 (SYT1_HUMAN).

Figure 1C shows positions of the mutated amino acids within a 3D structure of the SYT1 C2B domain. All five of the mutations map to the Ca^2+^ ion binding region of the C2B domain. Met303 packs into the interior of the domain against Ile374 and the aliphatic portion of Lys301. Two mutations, Asp304Gly and Asp366Glu occur in residues that directly contact both bound Ca^2+^ ions. Ile368 plays a critical role in the Ca^2+^-dependent penetration of SYT1 into the lipid membrane, and is thus central to the functional role of the protein (Paddock *et al.*, 2011). A hydrophilic residue at this position (Ile368Thr) is incompatible with insertion into the hydrophobic interior of the plasma membrane. Asn371 helps to define both the conformation of one of the Ca^2+^ binding loops and the position of the Ca^2+^ residue forming a ligand at Asp366.

To examine whether patient-identified mutations affect SYT1 structure, we performed molecular modelling and molecular dynamics simulations of the C2B domain. The average RMSD of each mutant model, which measures the divergence of the mutant protein structure from its initial structure over the course of the simulation, revealed that structural transitions occurred in the C2B domain incorporating M303K, which did not occur in the wild-type protein (Fig. 1D and Supplementary Fig. 1A). While most mutations did not affect the Ca^2+^-binding ability of the C2B domain, the average percentage of time bound for both Ca^2+^ ions across the simulation was significantly lower for D304G compared to wild-type (Fig. 1E, *P = *0.0028 Ca^2+ ^1, *P = *0.0001 Ca^2+^ 2; D304G versus wild-type, two-way ANOVA with Dunnett’s multiple comparison test). Therefore, these simulations provide evidence that *SYT1* mutations may alter the structure of the C2B domain and could thus be expected to have deleterious impact on SYT1 function.

### Case histories

For case history details see Table 1.

#### General health

Family histories, pregnancies and birth histories were unremarkable. No patient required neonatal resuscitation or intensive care. Congenital anomalies were absent, with exception of bilateral talipes in one individual and atrial septal defect (spontaneous closure) in another. Physical health during infancy and childhood was generally good. Feeding difficulties were reported for three children (difficulty chewing and swallowing solids), and gastro-oesophageal reflux was diagnosed in four. Central sleep apnoea was a feature in three patients, requiring supplemental oxygen during infancy but resolving by early childhood. Medical complications have been observed for the two individuals currently over the age of 10 years. Patient 1 (I368T) presented at age 11 years with paroxysmal episodes of cyanosis with an altered respiratory pattern, diagnosed on sleep study as hyperventilation-triggered apnoeas due to hypocarbia. Subsequent treatment with iron, clonidine and intermittent oxygen led to significant improvement of these episodes. Patient 3 (D304G) developed severe gastro-oesophageal reflux during his teenage years associated with food refusal, and also progressive lower limb contractures and scoliosis (surgically managed with poor outcome of reduced mobility).

#### Physical examination

Physical examination did not identify any consistent congenital abnormalities. Growth parameters indicated linear growth within the normal range. Notably, orbitofrontal circumferences were in the normal range (5th to 75th percentiles) and maintained during childhood. Comparison between photographs and clinical genetics evaluations suggested facial similarities between individuals, namely a prominent high forehead with V-shaped hairline, horizontal low-set eyebrows, mild epicanthus, almond-shaped eyes, fine facial features with short nose and prominent nasal tip, smooth philtrum and thin upper lip (Supplementary Fig. 2).

#### Neurological symptoms

The earliest sign of potential neurological impairment was infantile hypotonia, which was universal within the cohort. Patients were described as under-reactive to stimulation during the first year of life. Another common early feature was ophthalmic abnormality (strabismus in six cases, nystagmus in five cases) with poor visual attention and central visual impairment reported as a frequent later feature.

Post-infantile dystonic and hyperkinetic involuntary movement abnormalities currently affect four patients, with mutations I368T (two of four cases) and N371K (two of two cases). Symptom severity ranges from dystonic posturing and mild chorea to severe mixed movement disorder with vocal dystonia and ballismus. Illustrative videos are provided in the Supplementary material. A third case of I368T (Patient 10, currently age 3 years) demonstrates repetitive leg movements, stereotypies (hand clapping, throwing body backward) and possible lower limb dystonia, suggestive of evolving movement disorder with similarity to older patients with the same mutation. Three patients with mutation D366E, one patient with mutation D304G and one patient with I368T are not reported to have involuntary movements, but do manifest stereotypies such as repetitive leg kicking, finger chewing, object mouthing and head or chest tapping. One individual (M303K) is reported to have ataxia and impaired fine motor abilities, but no other movement abnormalities. No individual within the cohort has been diagnosed with a seizure disorder.

#### Neurodevelopment

Motor delay is reported to be mild in one individual (M303K), but is severe in the remainder (age of sitting independently 13 months to 4 years, one individual not yet sitting independently at age 4 years; age of walking independently 2–10 years, seven individuals not yet walking independently at ages 3–21 years). Speech and language skills are severely to profoundly impaired in 10 of 11 patients, with nine individuals using no words. However, Patient 6 (M303K) is reported to use around 50 words at the age of 7 years, indicating a milder degree of intellectual disability.

For all patients, behavioural disturbance is a major contributor to impairment and familial distress. Parents report a characteristic alternating pattern of switching between calm and excited or agitated phases, without apparent external triggers. During agitated phases, which can last between minutes and days, common problems include increased involuntary movements, screaming episodes, chest-beating, mouthing objects, chewing on fingers or hands, and minor self-injury. Impaired social development is also a common feature. Six of eleven individuals are reported to show no eye contact or poor eye contact, with limited interest in social interactions and absence of normal imitative behaviours. However, others are described as generally happy and socially engaged, except during episodes of agitation. Sleep disturbance is a major feature in at least seven patients, with frequent night waking and difficulty returning to sleep persisting to late childhood.

#### Treatment histories

A wide range of anti-epileptic treatments have been trialled (in view of EEG abnormality and severe neurodevelopmental impairment) including carbamazepine, sodium valproate, lamotrigine, leviteracetam, ethosuximide and ketogenic diet. Beneficial effects of these interventions have not been reported by clinicians or families, and sedation has been a frequent side-effect.

The two oldest patients have both been prescribed clonidine, and this was found to be beneficial in reducing sleep disorder and hyperventilation-induced cyanotic episodes.

We report our experience with treating Patient 1 (I368T) with pramipexole, a dopamine agonist with affinity for D_2_, D_3_ and D_4_ receptors, which is an established treatment option for parkinsonian movement disorders in adults and children. In view of functional evidence that the patient’s mutation alters the kinetics of neurotransmitter release, we hypothesized that a drug that amplifies post-synaptic function could potentially enhance synchronous neurotransmission. SYT1 is the major synaptotagmin isoform in the basal ganglia, where it plays an essential role in regulating calcium-dependent axonal dopamine release (Mendez *et al.*, 2011). Thus it was theorized that circumventing the impact of reduced dopaminergic release in the basal ganglia might potentially have a beneficial effect on striatal function and involuntary movements. Patient 1 has been treated daily with pramipexole for ∼3 years, with clinician-observed and parent-reported reduction in severity of movement disorder, reduced frequency and severity of agitated and self-injurious behaviours, and increased responsiveness to social and environmental stimuli. EEG abnormalities have also lessened. Based on a single patient open-label treatment experience we cannot conclude whether these improvements reflect a true response to medication or a coincident progression of the natural history of the patient’s disorder.

#### Neuroimaging and electrophysiology

All patients underwent cranial MRI on at least one occasion during diagnostic evaluations. Brain structure and qualitative assessment of maturation were reported as normal in seven cases. Delayed maturation was noted in one case. For Patient 7 (N371K), MRI at 6 months was reported to be normal but at 25 months of age periventricular white matter changes of uncertain significance were noted (Supplementary Fig. 3). For Patient 8 (D366E), MRI brain in infancy identified a choroid plexus haemorrhage, and a repeat MRI brain at 3 years of age was normal. For Patient 11 (I368T), MRI at 1 year showed mild generalized prominence of extra-axial CSF and patchy increased T_2_ signal in periventricular white matter of uncertain significance. No striatal or thalamic pathology or volume loss was reported for any case. Magnetic resonance spectroscopy was carried out for two cases and was normal in both.

**Figure 2:**
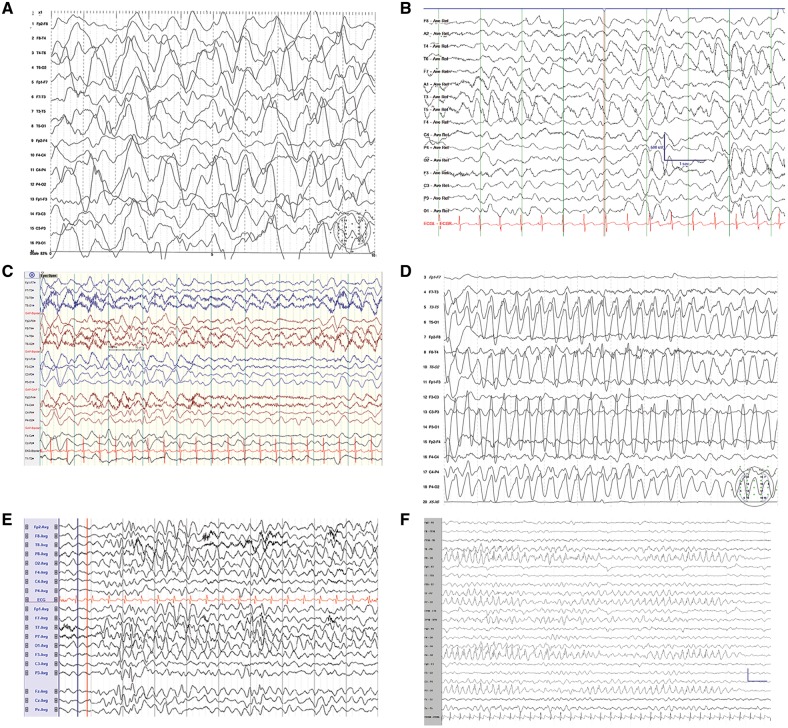
***SYT1 de novo* mutations are associated with low frequency oscillation bursts on EEG.** Clinical EEG acquired for 6 of 11 patients with *SYT1* mutations during early childhood. EEG for three patients with recurrent mutation SYT1 I368T: (**A**) Patient 2, age 8 months. (**B**) Patient 1, age 2 years. (**C**) Patient 10, age 3 years. EEG for patients with other mutations: (**D**) Patient 4, mutation SYT1 D366E, age 2 years. (**E**) Patient 5, mutation SYT1 N371K, age 2 years. (**F**) Patient 7, SYT1 N371K, age 3 years. Scale: *x* = 1 s, *y* = 600 µV.

All patients had clinical EEG recorded on at least one occasion during diagnostic evaluations, with abnormalities noted in all (Fig. 2). In all but one case, normal variable features of background activity were absent, and recordings were dominated by symmetrical bursts of high amplitude low frequency synchronous activity. The frequency of this slow wave activity varied with age (<1 year: frequency 1–3/s; 2–4 years: frequency 2.5–4/s; ≥8 years: frequency 5–6/s). Despite the absence of overt seizures, additional epileptiform abnormalities were reported in five individuals. These features include multifocal spikes, isolated spike bursts, isolated sharp waves and generalized spike wave discharges, most often with parietal-occipital maxima. For patients with multiple recordings available at different ages during childhood, epileptiform features decline with age, and some age-appropriate rhythms are observable by late childhood. Patient 9 (D366E) had a single EEG recording at 12 months of age, at which point background activity was reported to be normal, with the presence of rare low amplitude spikes from the right occipital region during drowsiness.

### Effect of *SYT1* mutations on the expression and trafficking of SYT1 protein

**Figure 3:**
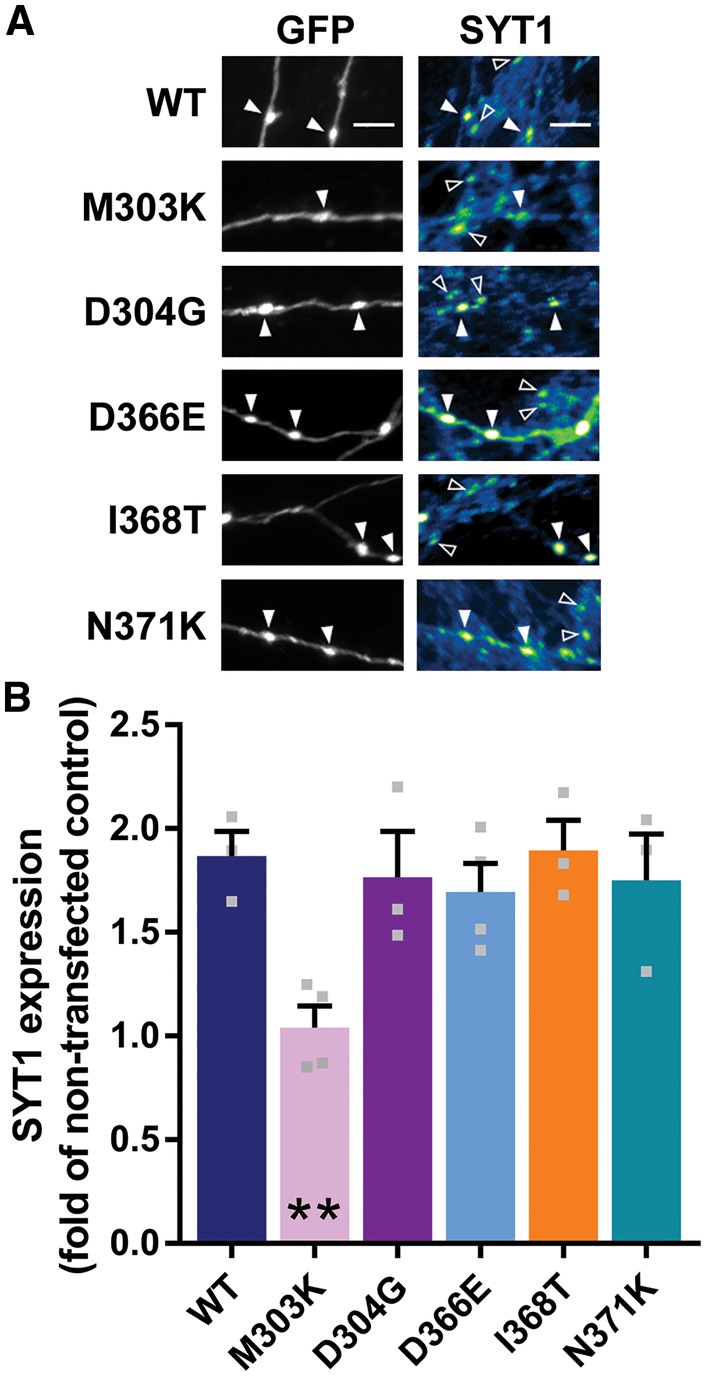
**SYT1 mutants, except M303K, are expressed as efficiently as wild-type protein.** Cultured hippocampal neurons were transfected with SYT1 variants. (**A**) Representative images of neurons transfected with SYT1 variants (tagged with pHluorin, a variant of GFP), fixed at rest and immunolabelled for GFP and SYT1. Greyscale panels (*left*) highlight transfected neurons (GFP), and false colour panels (*right*) display SYT1 immunofluorescence staining, with warmer colours indicating more intense staining. Arrowheads highlight transfected (filled) and non-transfected (open) nerve terminals. Scale bar = 5 μm. (**B**) Bar graph shows SYT1 immunofluorescence intensity in transfected neurons relative to non-transfected neurons in the same field of view. Data displayed as mean ± SEM, *n* = 3–4. **P < 0.01 compared to wild-type, one-way ANOVA with Dunnett’s multiple comparison test.

We first assessed whether mutations in SYT1 affected the ability of the protein to be expressed or targeted to central nerve terminals. Cultured hippocampal neurons were transfected with SYT1 variants and then immunolabelled for both EGFP (to identify transfected neurons) and SYT1 (Fig. 3A). This allows the level of expression of SYT1 variants to be determined by comparing to non-transfected cells in the same field of view.

Most SYT1 variants were expressed to equivalent levels as wild-type SYT1 (SYT1^WT^), with total SYT1 levels approximately double (1.7–1.9-fold) that of non-transfected cells (Fig. 3B). However, M303K displayed a significantly lower expression level than SYT1^WT^ (Fig. 3B, *P = *0.0088 compared to SYT1^WT^, one-way ANOVA with Dunnett’s multiple comparison test). Thus, all SYT1 variants, except SYT1^M303K^, were expressed in neurons at an approximately equal proportion to that of endogenous SYT1, effectively mimicking the heterozygous nature of the clinical cases.

**Figure 4:**
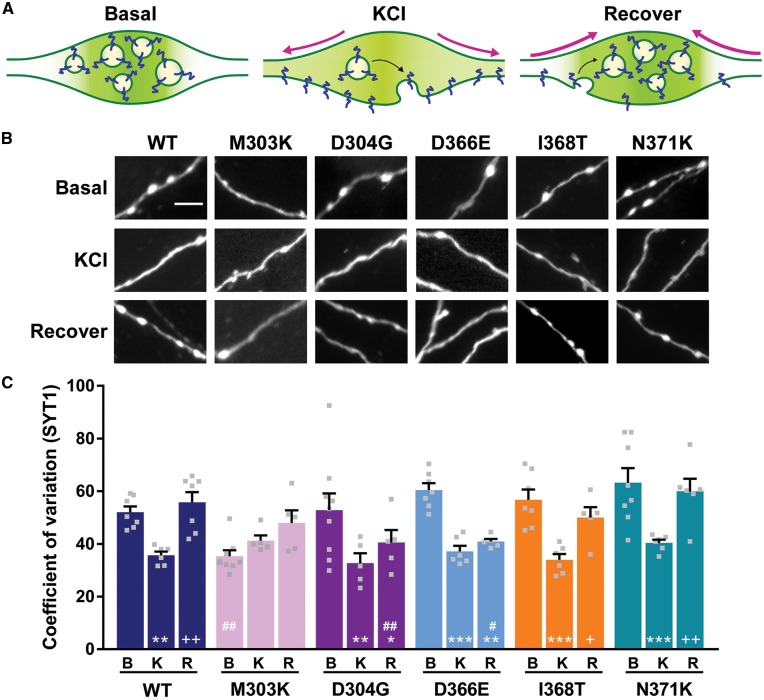
**SYT1 variants display mutation-specific defects in trafficking.** Cultured hippocampal neurons transfected with SYT1 variants were fixed at rest (basal; B), or immediately after 30 s incubation with 50 mM KCl (KCl; K), or after 2.5 min recovery in standard saline buffer following 30 s depolarization with 50 mM KCl (recover; R). All steps were performed at 37°C. (**A**) Diagram showing the localization of synaptic vesicle proteins (dark blue) in a presynaptic terminal at rest (*left*, basal), following stimulation (*middle*, KCl), and after recovery (*right*, recover). Colour intensity of background in presynaptic terminal represents fluorescence intensity of labelled proteins. Arrows (pink) indicate direction of change in protein localization and fluorescence signal. (**B**) Representative images of neurons transfected with SYT1-pHluorin variants, fixed and immunolabelled for GFP. Scale bar = 5 μm. (**C**) The distribution of fluorescence intensity along neurites determined by CV analysis, where a high CV equates to a punctate localization, indicative of targeting to presynaptic terminals. Data is mean CV ± SEM, *n* = 5–9. ^#^*P < *0.05, ^##^*P < *0.01 compared to wild-type within same condition, two-way ANOVA with Dunnett’s multiple comparison test. *P < 0.05, **P < 0.01, ***P < 0.001 compared to basal; ^+^*P < *0.05, ^++^*P < *0.01 compared to KCl; all by two-way ANOVA with Tukey’s multiple comparison test.

We next determined how efficiently SYT1 variants were targeted to nerve terminals. Coefficient of variation (CV) analysis was used to measure the localization of SYT1, where a high CV equates to a punctate distribution of fluorescence intensity, indicative of efficient localization to synaptic vesicles at nerve terminals, while a low CV equates to a diffuse distribution of fluorescence, indicative of the protein being more widely distributed throughout the axon (Lyles *et al.*, 2006; Gordon and Cousin, 2013). All SYT1 variants, with the exception of SYT1^M303K^, targeted efficiently to nerve terminals at rest (Fig. 4B). SYT1^M303K^ displayed a more diffuse localization than SYT1^WT^, and had a significantly lower CV (Fig. 4C, *P = *0.0064 compared to basal SYT1^WT^, two-way ANOVA with Dunnett’s multiple comparison test). Therefore, SYT1^M303K^ is dysfunctional in its level of expression and retention at nerve terminals.

We next determined whether there were any global defects in the ability of SYT1 variants to be mobilized upon neuronal activity. During depolarization-induced Ca^2+^ influx, synaptic vesicles undergo exocytosis and SYT1 escapes the nerve terminal and is redistributed towards the periactive zone. SYT1 is subsequently retrieved from the plasma membrane by synaptic vesicle endocytosis (Fig. 4A). The activity-dependent change in the fluorescence profile of neurons expressing each SYT1 variant was therefore monitored before, during or after depolarization with 50 mM KCl.

We first examined the effect of the identified mutations on the activity-dependent redistribution of SYT1 fluorescence out of nerve terminals during synaptic vesicle exocytosis (Fig. 4B). This was achieved by monitoring the decrease in CV on stimulation. As expected, CV immediately after stimulation was significantly lower than CV at rest for SYT1^WT^, indicating that evoked synaptic vesicle exocytosis had occurred. This was also the case for all SYT1 variants (Fig. 4C, basal versus KCl, wild-type *P = *0.0098; D304G *P = *0.0012; D366E *P = *0.0001; I368T *P = *0.0002; N371K *P = *0.0001; two-way ANOVA with Tukey’s multiple comparison test), with the exception of SYT1^M303K^, which was already mislocalized before stimulation.

Next, we assessed whether these mutations affected the re-enrichment of SYT1 at nerve terminals, which is reliant on efficient synaptic vesicle endocytosis. Neurons were depolarized with 50 mM KCl and allowed to recover in standard saline buffer for 2.5 min, and the fluorescence profile of each SYT1 variant was again measured. This recovery period is sufficient for endocytosis to take place and for the reclustering of synaptic vesicles, and thus re-enrichment of SYT1^WT^, at nerve terminals (Fig. 4B and C, KCl versus recover, wild-type *P = *0.0011; two-way ANOVA with Tukey’s multiple comparison test). SYT1^I368T^ and SYT1^N371K^ were also re-enriched at nerve terminals during the recovery period (Fig. 4B and C, KCl versus recover, I368T *P = *0.0234; N371K *P = *0.0024; two-way ANOVA with Tukey’s multiple comparison test), displaying a similar localization profile to SYT1^WT^. In contrast, both SYT1^D304G^ and SYT1^D366E^ remained diffusely localized after stimulation, and the CV of these variants remained significantly lower than that at rest (Fig. 4B and C, basal versus recover, D304G *P = *0.0146; D366E *P = *0.0028; two-way ANOVA with Tukey’s multiple comparison test) and thus these variants did not efficiently relocalize to nerve terminals. This suggests that the retrieval of SYT1^D304G^ and SYT1^D366E^ from the plasma membrane was arrested, resultant from either a generalized defect in endocytosis, or a specific failure of these variants to be recognized by the endocytic machinery.

#### Effect of *SYT1* mutations on the rate of exocytosis

The protein localization assay revealed that synaptic vesicle exocytosis proceeds in the presence SYT1 mutants; however, it provides no information regarding the kinetics of exocytosis. To examine this in real time, we used the genetically-encoded reporter SYT1-pHluorin (a pH-sensitive form of EGFP, fused to the lumenal domain of SYT1), which was expressed in cultured hippocampal neurons. pHluorin fluorescence is quenched inside the acidic lumen of synaptic vesicles but fluorescence increases upon exposure to the neutral extracellular medium during exocytosis. Fluorescence is quenched again following endocytosis as nascent synaptic vesicles are re-acidified. Exocytosis can be investigated specifically by arresting synaptic vesicle acidification with the V-type ATPase inhibitor bafilomycin A1. This permits a quantification of both the rate and extent of synaptic vesicle fusion during neuronal activity.

**Figure 5:**
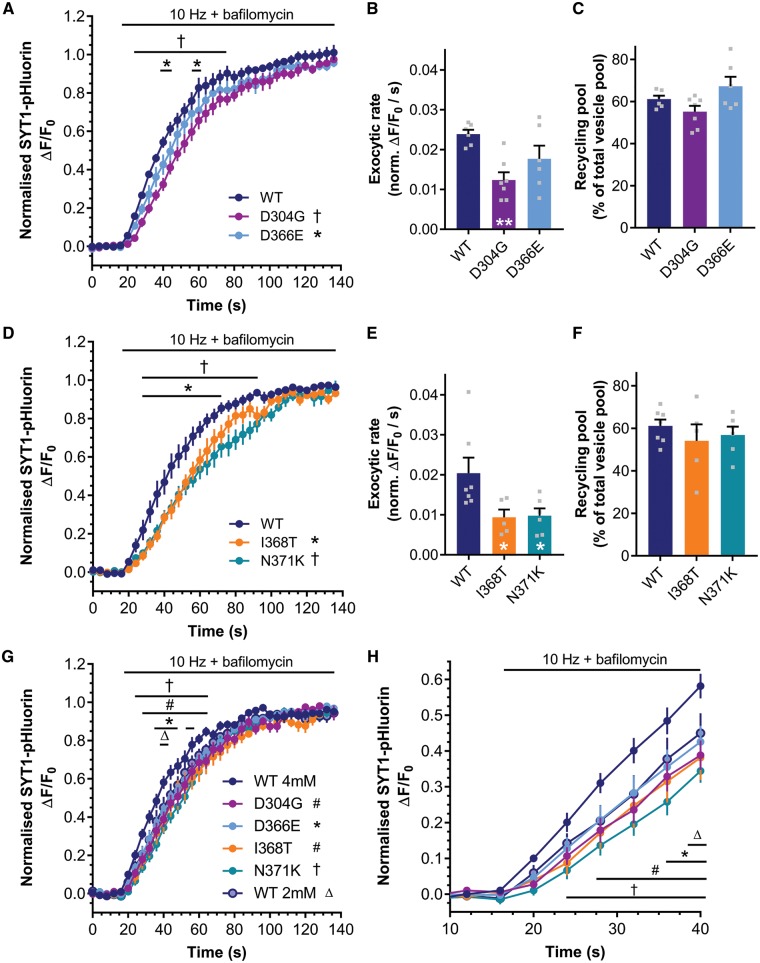
**SYT1 mutants slow evoked exocytosis.** Hippocampal neurons transfected with SYT1-pHluorin variants were stimulated with a train of 1200 action potentials at 10 Hz, in the presence of 1 µM bafilomycin A1 to block vesicle reacidification. (**A** and **D**) Time course of mean ΔF/F_0_ of SYT1-pHluorin variants normalized to stimulation peak. (**B** and **E**) Mean initial rate of exocytosis calculated from linear fit of ΔF/F_0_ per second over the first 20 s of stimulation (normalized to stimulation peak). (**C** and **F**) Total vesicle pool mobilized by 1200 action potentials at 10 Hz, normalized to NH_4_Cl peak. (**A**) ^†^*P < *0.05 for SYT1^D304G^-pH and *P < 0.05 for SYT1^D366E^-pH against SYT1^WT^-pH over time indicated by bar [wild-type (WT) *n = *6, D304G *n = *7, D366E *n = *6, repeated measures ANOVA with Dunnett’s multiple comparisons test]. (**B**) **P < 0.01 (*n* as in **A**, one-way ANOVA versus wild-type with Dunnett’s multiple comparisons test). (**C**) Not significant by one-way ANOVA, *n* as in **A**. (D) *P < 0.05 for SYT1^I368T^-pH and ^†^*P < *0.05 for SYT1^N371K^-pH against SYT1^WT^-pH over time indicated by bar (wild-type *n = *7, I368T *n = *5, N371K *n = *6, repeated measures ANOVA with Dunnett’s multiple comparisons test). (**E**) *P < 0.05 (*n* as in **D**, one-way ANOVA versus wild-type with Dunnett’s multiple comparison test). (**F**) Not significant by one-way ANOVA, *n* as in **D**. (**G** and **H**) Hippocampal neurons transfected with SYT1-pHluorin variants were perfused with high (4 mM) Ca^2+^ buffer (wild-type 4 mM, D304G, D366E, I368T, N371K) or normal Ca^2+^ buffer (wild-type 2 mM), and were stimulated with a train of 1200 action potentials at 10 Hz, in the presence of 1 µM bafilomycin A1 to block synaptic vesicle re-acidification. (**G**) Time course of mean ΔF/F_0_ of SYT1-pHluorin variants normalized to stimulation peak. *P < *0.05 for SYT1^D304G #^*P < *0.05 for SYT1^I368T^-pH, *P < 0.05 for SYT1^D366E^-pH, and ^†^*P < *0.05 for SYT1^N371K^-pH, against 4 mM SYT1^WT^-pH over time indicated by bar (*n* = 8 for 4 mM wild-type, D366E, I368T; *n = *7 for 2 mM wild-type, D304G, N371K, repeated measures ANOVA with Tukey’s multiple comparisons test). (**H**) Same data as in **G**, but cut-off at 40 s for clarity. All data represented as mean ± SEM.

Neurons transfected with SYT1-pHluorin variants were stimulated with a train of 1200 action potentials at 10 Hz in the presence of 1 µM bafilomycin A1, and both the extent and rate of the evoked fluorescence increase was monitored. When the aspartate SYT1 variants SYT1^D304G^ and SYT1^D366E^ were examined, evoked exocytosis was able to proceed, as evidenced by the increase in pHluorin fluorescence upon stimulation (Fig. 5A). The proportion of total synaptic vesicles that underwent fusion (i.e. the recycling pool of vesicles) was also unaffected by these SYT1 variants (Fig. 5C). However, the presence of SYT1^D304G^ and SYT1^D366E^ resulted in a slowing of exocytosis compared to SYT1^WT^ (Fig. 5A, *P < *0.05 SYT1^D304G^ and SYT1^D366E^ versus SYT1^WT^, repeated measures ANOVA with Dunnett’s multiple comparison test). The severe effect of SYT1^D304G^ was reflected by a reduction in the initial rate of exocytosis over the first 20 s of stimulation (Fig. 5B, *P = *0.0042 SYT1^D304G^ versus SYT1^WT^, one-way ANOVA with Dunnett’s multiple comparison test). In contrast, SYT1^D366E^ had a milder effect (Fig. 5A) and did not significantly reduce the initial rate of exocytosis (Fig. 5B, *P = *0.1365 SYT1^D366E^ versus SYT1^WT^ one-way ANOVA with Dunnett’s multiple comparison test).

We next investigated the remaining non-aspartate SYT1 variants. Similarly to SYT1^D304G^, the recycling pool of vesicles was not affected by SYT1^I368T^ or SYT1^N371K^ (Fig. 5F), but exocytic rate was reduced in comparison to SYT1^WT^ (Fig. 5D, *P < *0.05 SYT1^I368T^ and SYT1^N371K^ versus SYT1^WT^, repeated measures ANOVA with Dunnett’s multiple comparison test; Fig. 5E, *P = *0.0388 SYT1^I368T^ and *P = *0.0362 SYT1^N371K^ versus SYT1^WT^, one-way ANOVA with Dunnett’s multiple comparison test). Mislocalization of SYT1^M303K^ precluded it from examination with this assay.

Since increased Ca^2+ ^influx can mitigate the clinical symptoms of similar mutations in the related gene *SYT2* (Herrmann *et al.*, 2014; Whittaker *et al.*, 2015), these exocytosis assays were repeated in the presence of increased extracellular Ca^2+^ (4 mM, in place of normal physiological 2 mM Ca^2+^), to determine if this could ameliorate SYT1 mutant-dependent slowing of exocytosis. At 4 mM Ca^2+^, exocytosis remained slower for SYT1 variants *in vitro* compared to SYT1^WT^ (Fig. 5G and H, *P < *0.05 SYT1^D304G^, SYT1^D366E^, SYT1^I368T^ and SYT1^N371K^ versus SYT1^WT 4 mM^, repeated measures ANOVA with Tukey’s multiple comparison test). Importantly, however, the exocytic rate with all SYT1 variants at 4 mM Ca^2+^ was restored to that of SYT1^WT^ at 2 mM Ca^2+^ (Fig. 5G and H, *P* > 0.05 SYT1^D304G^, SYT1^D366E^, SYT1^I368T^ and SYT1^N371K^ versus SYT1^WT 2 mM^, repeated measures ANOVA with Tukey’s multiple comparison test). Therefore, increasing extracellular Ca^2+^*in vitro* can normalize the rate of synaptic vesicle exocytosis back to physiological levels in neurons expressing SYT1 variants.

## Discussion

We report here the clinical characteristics of 11 patients with *de novo* missense mutations in *SYT1.* Mutations in this gene are associated with a recognizable neurodevelopmental phenotype comprising infantile hypotonia, ophthalmic abnormalities with delayed visual maturation, sleep disturbance, movement abnormalities, motor delay and intellectual disability. We have been struck by the similarity in behavioural features across this case series. Parents report an alternating, unpredictable pattern of activity, switching between calm and excited states without obvious provocation. These behavioural characteristics are independent of the severity of intellectual disability or presence of movement disorder, hence may be useful diagnostic markers.

Movement abnormalities are an important aspect of the condition, and a spectrum of severity has been observed within the case group. Dystonia, dyskinesia or hyperkinetic movement disorder has been diagnosed in four patients. Hence the presence of an involuntary movement disorder may be suggestive of *SYT1* mutation, but absence does not preclude this diagnosis. In other cases, review of parental video material has revealed less severe movement abnormalities, for example repetitive leg kicking and posturing, not reaching threshold for neurological classification. Motor stereotypies are common within the group: hand-biting and finger-chewing is a prominent stereotypy for the majority of individuals, whilst head-butting and chest-beating are also observed in some cases. Distinguishing these repetitive actions from true involuntary movements is not straightforward, requiring multiple observations in different settings and at different times of the day. Longitudinal data on this relatively young cohort will establish whether involuntary movement disorder emerges in a higher proportion of patients with time, and will clarify whether there is a predictable sequence of symptom evolution and resolution. Age is unlikely to be the only predictor of symptom severity since the age range of patients currently manifesting an involuntary movement disorder (3–14 years) is similar to the age range of currently unaffected patients (3–21 years).

Beyond prognostication, diagnosis of *SYT1*-associated neurodevelopmental disorder can have important treatment implications. No beneficial effect of anti-epileptic drug (AED) treatment has been observed on either neurodevelopmental outcome or electrophysiological abnormalities. In contrast, patients revealed side effects mainly consisting of sedation. Hence AED treatment for patients harbouring *SYT1* mutations should be considered with caution. We are encouraged by the potential benefits of the dopamine agonist pramipexole. Initiation of pramipexole in Patient 1 was associated with rapid and sustained reduction in involuntary movements and agitation; however, this treatment has yet to be trialled in a second patient.

We have collated clinical EEG data for all patients and found that electrophysiological abnormality is a consistent hallmark of *SYT1* mutation. Recordings are dominated by bursts of synchronous, slow wave, high voltage activity plus isolated epileptiform spike activity. The presence of typical EEG features will provide important supportive evidence for pathogenicity in future diagnostic evaluations. However, there is also electrophysiological variation between cases. Oscillatory bursts vary in durations, cycle frequency and cerebral distribution, and the extent and morphology of spike activity is also variable. Potential explanations for this variation include differences between patients in EEG recording conditions, for example medication status, use of melatonin during recordings, and arousal status. Age at clinical EEG recordings may also be an important factor, since we observe an increase in oscillatory frequency and reduction in spike activity with age. Another possible source of variation could be genotype-specific electrophysiological signatures. Although we surmise that all *SYT1* mutations disturb subcortical-cortical network properties, leading to unconstrained low frequency synchronous activity, the severity and clinical consequence of this disturbance may vary. These differences may arise from the specific impact of each mutation on synaptic physiology, for example whether the amino acid substitution disturbs calcium binding or membrane penetration, which will be characterized in future studies, and consequent influence of these mutations on synaptic vesicle dynamics and network activity.

The presence of recurrent *de novo* missense mutation associated with a specific disease is most commonly explained by specific dominant negative or gain-of-function effects, such as those in *FGFR3* causing achondroplasia (Bellus *et al.*, 1995). On the other hand, the presence of non-recurrent missense mutations can lead to a variety of loss- and gain-of-function effects, associated with phenotypic variability, as has been observed for *SCN8A* and *GABRG2* (Blanchard *et al.*, 2015; Warner *et al.*, 2016). To obtain further evidence for the pathogenicity of newly-identified mutations and explore physiological correlates of patients’ phenotypes, we introduced rat SYT1 mutants (equivalent to the five patient-identified mutations) into primary hippocampal cultures to screen for global defects in SYT1 functionality. SYT1^M303K^ is unique in that it was expressed at lower levels than SYT1^WT^, with reduced targeting to nerve terminals at rest as well as reduced somatic expression (Supplementary Fig. 4). Molecular modelling predicts that, of the mutations examined, M303K produces the greatest change to the structure of the C2B domain, potentially compromising the expression, stability or trafficking of this variant. This may limit deleterious dominant-negative effects or constitute loss of function in neurons, resulting in the individual harbouring this SYT1 variant displaying the mildest neurodevelopmental impairment within the cohort.

Further examination of mutants that displayed normal expression and localization profiles at rest, revealed that these were able to be mobilized upon depolarization, indicating that exocytosis was not abolished (indeed, cessation of exocytosis would be incompatible with life). Interestingly, SYT1 variants with mutations of Ca^2+^-binding residues were not retrieved following depolarization as efficiently as the wild-type protein, indicating that these residues might be important for either the specific trafficking of SYT1 back to vesicles or for facilitating synaptic vesicle endocytosis globally. Future studies will be required to tease apart these distinct pathways. This suggests that endocytic defects contribute to the pathophysiology of neurological dysfunction in individuals harbouring these mutations. Previous studies using multi-site C2A/C2B domain aspartate mutants [D230 232N in C2A in combination with D363, D365N in C2B (D365 in rat is equivalent to D366 in human)] revealed the importance of these Ca^2+^-binding residues for efficient endocytosis. Expression of this multi-site mutant in SYT1 knockout mouse neurons failed to rescue the kinetics of endocytosis back to wild-type levels (Yao *et al.*, 2011). However, mutation of D363, D365N in the C2B domain alone had no effect on the retrieval of SYT1 to synaptic vesicles following stimulation (Yao *et al.*, 2011). This may be a result of the nature of the mutations (D366/5E versus D365N), or the different stimuli used in these studies evoking different modes of endocytosis. Alternatively, endogenous wild-type SYT1 may be preferentially retrieved over aspartate mutant SYT1 in our culture system, while the D363, D365N SYT1 mutant was examined in neurons lacking endogenous SYT1. It will be important to ascertain in future work whether there is a stimulation threshold or stimulation intensity dependence for deficient retrieval of SYT1 C2B aspartate mutants.

SYT1 has well-defined functions in mediating evoked synchronous neurotransmitter release, both through its membrane-penetrating ability and interactions with fusogenic exocytic machinery (Martens *et al.*, 2007; Hui *et al.*, 2009; Zhou *et al.*, 2015, 2017). These functions are heavily dependent on the C2B domain (Mackler *et al.*, 2002; Nishiki and Augustine, 2004; Paddock *et al.*, 2011). All neurodevelopmental disease-associated variants of SYT1 that were correctly targeted to nerve terminals were observed to slow the rate of exocytosis, noting that our experimental paradigm does not allow distinction between synchronous and asynchronous release. The downstream neurophysiological impact of slowed exocytosis on postsynaptic activation, neural network activity and information processing is likely to be cell-type specific i.e. impact maximally in regions and neuronal subpopulations where other SYT isoforms are unavailable to compensate. Accordingly, SYT1 is the major isoform mediating terminal dopamine release in the midbrain, providing a potential explanation for involuntary movement disorders (Mendez *et al.*, 2011). Moreover, SYT1 expression in the neocortex increases during prenatal and early postnatal brain development, then plateaus and declines from mid-childhood (Kang *et al.*, 2011) (hbatlas.org), in line with our observations of the natural history of patients’ symptoms and electrophysiological phenotype.

We found preliminary evidence for genotype–phenotype correlation, both with regard to clinical presentation and impact on exocytosis *in vitro.* D366E has the mildest effect on exocytic rate; correspondingly, individuals harbouring the D366E variant manifest milder motor delay, devoid of early-onset movement disorder, but retaining characteristic EEG abnormalities and severe cognitive impairment. The milder effect of this mutant recapitulates work in *Drosophila*, where an equivalent syt variant was largely able to rescue synchronous release (Nishiki and Augustine, 2004). In contrast, mutations to the residue equivalent to D304 were only able to partially rescue neurotransmitter release (Nishiki and Augustine, 2004). Correspondingly, we observe a severe cognitive outcome and severe impairment in exocytic rate in association with the D304G variant. Both patients with N371K and two of four patients with I368T presented with early-onset dystonia and choreo-athetosis plus severe to profound intellectual disability. Both variants were observed to have a similarly severe impact on exocytic rate. Mutation at I368 has previously been shown to have a dominant-negative effect on neurotransmitter release by impeding membrane penetration (Paddock *et al.*, 2011). This is the first study to investigate mutation of N371, hence further investigation is required to define a molecular mechanism of action. Identification of additional patients and mutations, plus experimentally controlled data on electrophysiological characteristics and symptom correlates, are required to confirm and extend these observations.

Clinical symptoms in individuals harbouring mutations in the related gene, *SYT2*, can be improved by 3,4-diaminopyridine, a K^+^ channel blocker that increases Ca^2+^ influx into the nerve terminal during neuronal activity (Whittaker *et al.*, 2015). We hypothesized that increasing extracellular Ca^2+^ levels could ameliorate exocytic defects induced by SYT1 mutants. Importantly, we found that this intervention *in vitro* could nullify the effect of these variants, such that exocytosis proceeded at the wild-type rate at physiological Ca^2+^ concentrations. Further studies are required to investigate the impact of exocytic rate and its manipulation on neural circuit activity *in vitro* and *in vivo* model organisms, to inform potential therapeutic strategies for individuals with *SYT1*-assosciated neurodevelopmental disorder.

Several other genes involved in neurotransmitter release have been implicated in neurodevelopmental disorders. Mutations in *PRRT2*, which influences Ca^2+^-dependent synchronous release via interaction with SYT1 (Valtorta *et al.*, 2016), lead to benign infantile familial seizures, paroxysmal movement disorders and intellectual disability (Ebrahimi-Fakhari *et al.*, 2015). Mutations in the plasma membrane SNARE protein SNAP-25 result in epilepsy and intellectual disability (Hamdan *et al.*, 2017); these mutations occur in or near residues that bind directly to the C2B domain of SYT1 (Zhou *et al.*, 2017). Mutations in proteins that regulate endocytosis, such as synaptophysin and dynamin 1, have also been linked to neurodevelopmental disorders involving movement abnormalities and EEG disturbance (Ferguson *et al.*, 2007; Tarpey *et al.*, 2009; Gordon *et al.*, 2011; Kwon and Chapman, 2011; von Spiczak *et al.*, 2017). Intriguingly, a dominant-negative mutation in *UNC13A*, which accelerates synaptic vesicle fusion, is also associated with dyskinesia and intellectual disability, reinforcing the importance of tight regulation of the kinetics of neurotransmission (Lipstein *et al.*, 2017). Diagnosis of further patients with mutations in these genes, systematic characterization of phenotypes, and identification of additional disorders of synaptic vesicle cycling will determine the extent of convergence between presynaptic pathway-associated disorders.

In summary, we report the identified mutations and clinical phenotypes for 11 individuals with *SYT1* mutation, and assessment of the impact of these mutations on SYT1 functionality. Collectively, these data confirm that *SYT1* mutation is associated with a recurrent neurodevelopmental disorder. We found that each of these mutations detrimentally affect either the expression and localization, or functionality of SYT1. Variation in clinical phenotype severity, in combination with differential effects of SYT1 mutants *in vitro*, points toward mutation-specific mechanisms underlying neurological dysfunction. Characterization of this new disorder highlights the key roles of SYT1 in presynaptic vesicle dynamics and the developmental emergence of motor control and cognitive abilities.

## Supplementary Material

awy209_Supplementary_Materials

## Abbreviation

CV: coefficient of variation
