# Supplementary material for: SYT1-associated neurodevelopmental disorder: a case series
Source: Brain. 2018 Aug 13;141(9):2576–91. doi: 10.1093/brain/awy209 (PMC6113648; doi:10.1093/brain/awy209)
Supplement: awy209_Supplementary_Materials [file awy209_supplementary_materials.zip › awy209-suppl_data/190702_Kahn et al_Brain_supp_awz209.pdf]

Supplementary material

Supplementary Figure 1. Mossy fibre sprouting and hippocampal damage

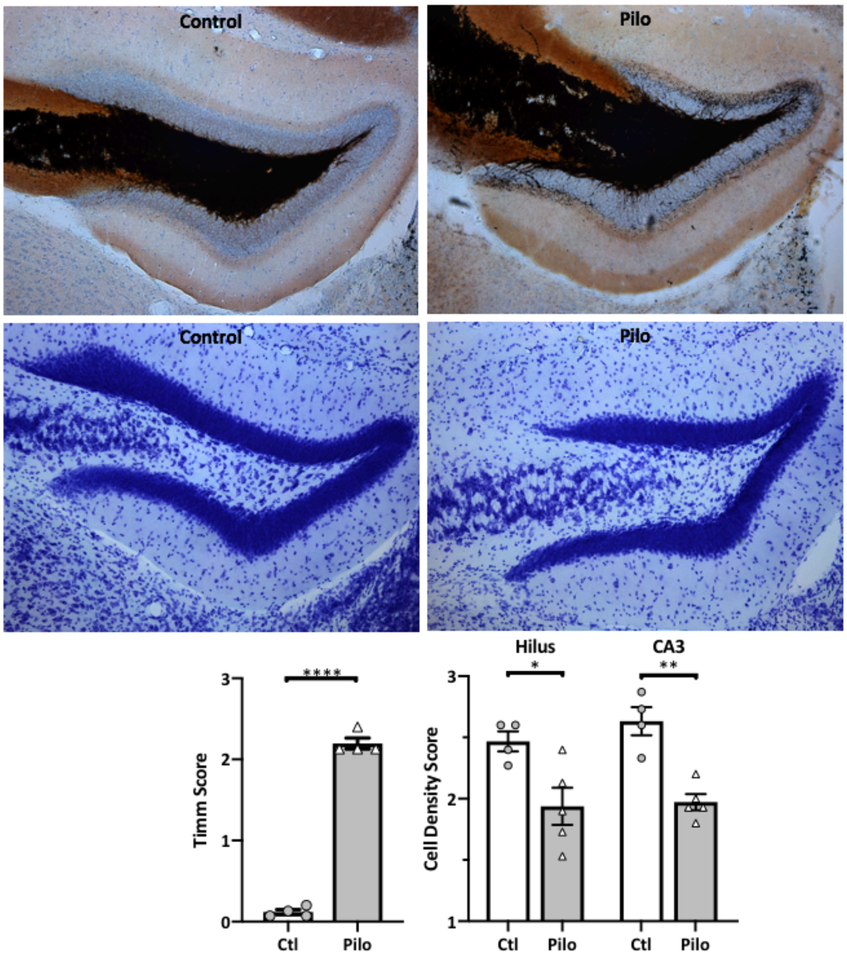

To assess mossy fibre sprouting and hippocampal damage in pilocarpine-treated mice, we performed Timm's (top) and Nissl staining (middle), respectively. Sections were assessed semi-quantitatively by five investigators who were blind to the experimental treatment. Timm's stain was quantified (bottom left) using a modified Tauck and Nadler scale, where zero indicated little or no Timm staining in the granule cell or inner molecular layer; 1 signified mild, sparse staining in inner molecular layer; 2 indicated moderate, patchy staining through the inner molecular layer; and 3 denoted a continuous band of staining throughout the inner molecular layer. Damage was assessed via cell density (bottom right). Cell density in the hilar and CA3 region were quantified where 3 indicated dense, intact cell bodies; 2 signified moderate cell density; and 1 denoted sparse, virtually absent cell bodies. Investigators' scores were averaged together for each section. Statistical comparisons were Student's *t*-tests. Data are represented as mean ± SEM. Each circle represents the average of 2-3 sections per animal. *$p<0.05$, **$p<0.01$, ***$p<0.001$, ****$p<0.0001$.

Supplementary Figure 2. Viral expression characterization

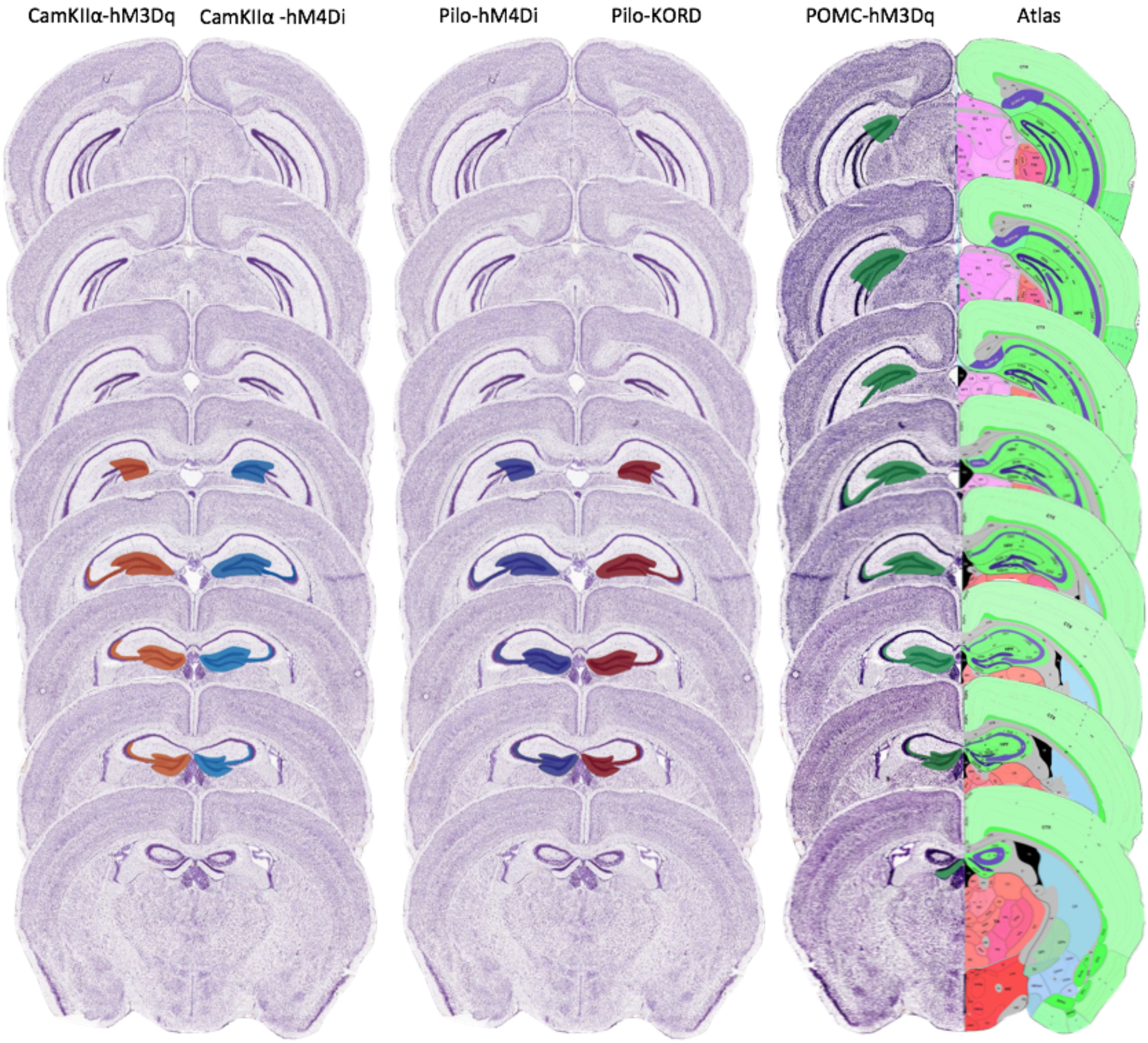

Brain atlas images (from *The Allen Mouse Brain Atlas,* https://mouse.brain-map.org/, Lein *et al*., 2007, *Nature*) are overlaid with the largest spread of fluorescent reporter across the rostral-caudal hippocampal axis. Each hemisphere represents the spread in one control/disease state + DREADD viral vector. Rostral to caudal brain atlas locations from Bregma are as follows: -1.06 mm, -1.34 mm, -1.70 mm, -2.18 mm, -2.46 mm, -2.80 mm, -3.08 mm, -3.28 mm.

Supplementary Figure 3. Seizure clusters in pilocarpine mice

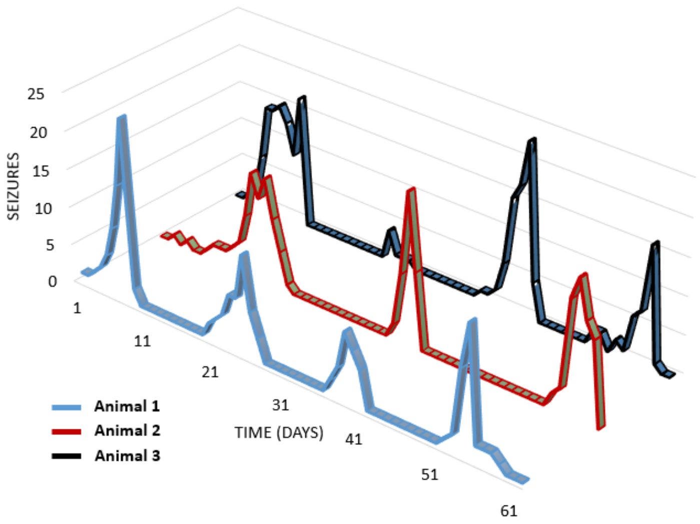

Total seizures from all mice: 557
Seizures with a behavioral component: 557
Mean Cluster Duration: 6.3 (SD: 2.3) days
Mean Inter-Cluster Duration: 9.6 (SD:3.3) days
Mean # of clusters per month: 1.61
Average Cluster Peak: 15.3 seizures (SD:5.5)
Average # of Seizures per Cluster: 49.7 (SD:22.2)

Pilocarpine treated mice were implanted with EEG depth electrodes one week post-status epilepticus and were allowed to recover for one week. They were recorded continuously for two months to determine the duration and frequency of seizure clusters. Seizure events were monitored using video behavioural monitoring and depth electrode EEG recordings. EEG recordings were performed using a Stellate-Harmonie (Stellate Inc., Montreal, Canada) 16-bit, 32 channel digital Video-EEG machine, sampling at 200 Hz. Cortical electrodes consisted of self-tapping screws and hippocampal electrodes made of bipolar 0.005 inch stainless steel wire. The depth electrode was placed into CA1 of the right hippocampus. Cortical electrodes were placed directly in front of the Bregma suture on both side of the midline. All electrodes were held in a six-pin pedestal. Ground and reference electrodes were placed directly behind the Lambda suture on either side of the midline. The pedestal of electrodes and screws were secured with dental cement. EEG tracings were analysed with custom Matlab code and reviewed to confirm electrographic seizures. If a seizure was identified, the simultaneous occurrence of a behavioural seizure was examined. **Left.** Representative graph of three animals showing the total number of seizure events each animal experienced each day over the course of the monitoring period. **Right.** Table of statistics for the monitored animals.

Supplementary Figure 4. Voltage sensitive dye recordings reveal DG hyperactivity in epileptic slices

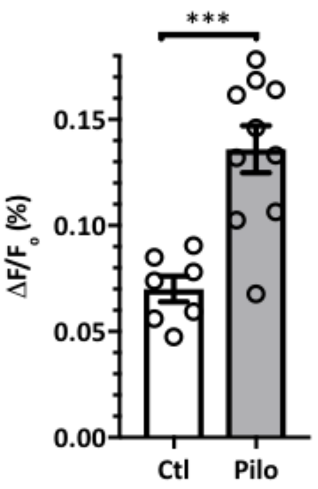

To examine differences in DG excitability between the normal and disease state, we analysed the voltage sensitive dye recordings in aCSF of the epileptic and control slices from Figures 6 and 8. Epileptic slices (n=10 animals) display a significant increase in activity in the DGC and molecular layers compared to control slices (n=7 animals) in response to electrical stimulation in the molecular layer ($t_{13.4}$=5.224, p=0.001, Welch's t-test). Data are represented as mean ± SEM. Each circle represents an animal (each circle indicates the mean of 2-3 slices for a given animal). *p<0.05, **p<0.01, ***p<0.001.

Supplementary Figure 5. Circuit activity-behavioural performance tuning curve

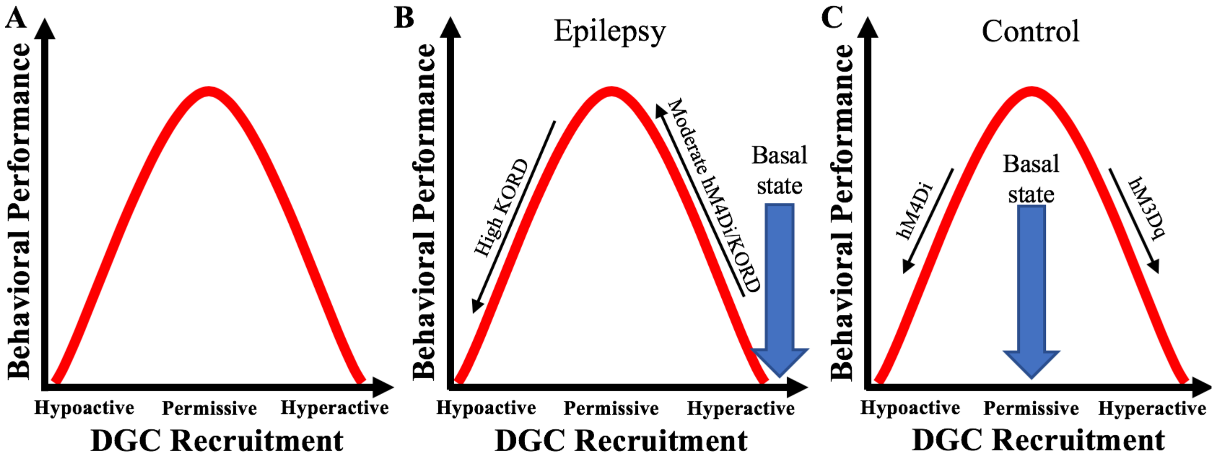

A. Schematic of the hypothesized circuit activity-behavioural performance tuning curve. Our data suggest that there is a relationship between circuit activity and cognitive performance (Fig 2, 3). DGC hyperactivity masks the circuit's informational output in noise, while too little DGC activity fails to code sufficient information. Rather, there is a permissive window of activity in which the circuit can effect successful behavioural outcomes. **B.** In epileptic animals, the DG undergoes many structural changes that aggregate to produce a hyperactive circuit, which fails to support DG-dependent tasks. However, with "moderate" inhibitory DREADD recruitment (i.e., treatment with lower levels of DREADD ligands CNO or SB), DGC activity is constrained such that the circuit operates within a permissive activity window that supports SOR performance. Overly recruiting the inhibitory KORD with a higher dose of the SB ligand makes the circuit overly quiescent, forcing the circuit too far to the "left," again disrupting behavioural performance. **C.** Control animals have a properly tuned DG such that manipulating DGC recruitment to be hypoactive with hM4Di or hyperactive with hM3Dq disrupts DG-mediated behaviour.

Supplementary Figure 6. Seizure detection during behaviour

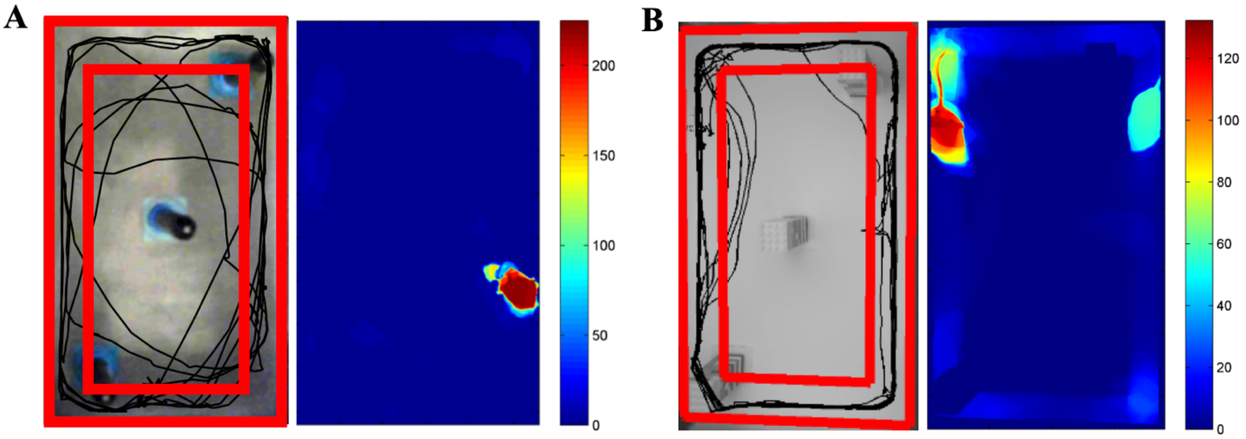

To ensure that behavioural outcomes were not a function of seizure activity, we performed a positive control for how an epileptic animal would behave when seizing. We monitored epileptic animals daily with video behavioural monitoring and depth electrode EEG recording until they were in an identifiable seizure cluster (2-4 days of at least 3 seizures per day). The mice became dramatically more anxious and aggressive with the handler when they were mid-cluster. We then tested them on the SOR task. They showed prolonged freezing (>2 min) while in the testing environment, quantified with locomotion analysis and video verification of freezing (vs grooming). Thus, seizure-related events were accompanied with observable behavioural features that allowed us to identify and exclude affected subjects from our analyses. **A.** (left) Locomotion tracking of an animal experiencing EEG-identified seizures. (right) Heat map of the cumulative time (s) the animal spent in each location during the session. **B.** Locomotion tracking and heat map of one of the animals excluded from behavioural analysis due to observed seizure-related behaviour during training. Notably, this animal expressed CamKIIα-KORD bilaterally in its dorsal DGs and was treated with 1.5 mg/kg SB 1 hour prior to this session. This subject, along with four CamKIIα-hM4Di-expressing mice treated with 1.5 mg/kg CNO who similarly displayed extended freezing or forelimb clonus, suggest that the dorsally expressed inhibitory DREADDs were not sufficient to suppress seizure events.

Supplementary Figure 7. High KORD recruitment does not suppress seizure clusters

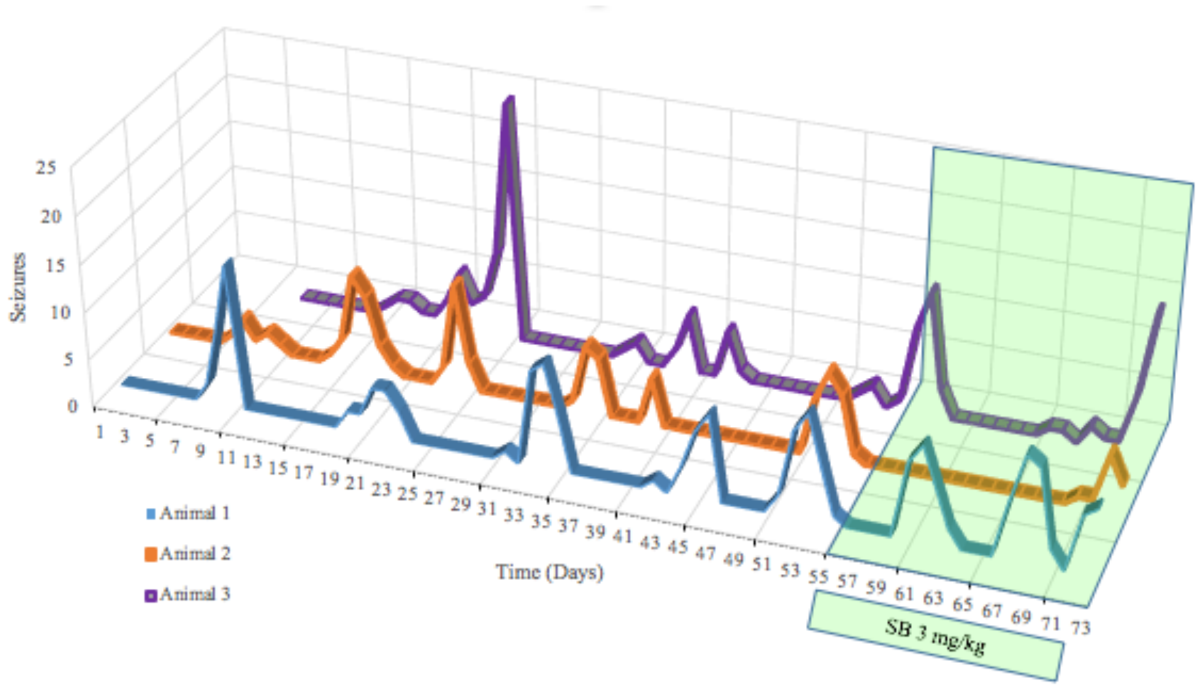

Pilocarpine treated animals were injected bilaterally with the CamKIIα-KORD and were implanted with EEG depth electrodes. They were monitored continually for up to 73 days. After a baseline was established, the mice received a daily subcutaneous injection of 3 mg/kg SB (the "high" SB dose used in Fig. 3B). During the 19 days of treatment (shaded green), mice continued to display seizure clusters.

Supplementary Figure 8. Locomotion Analysis

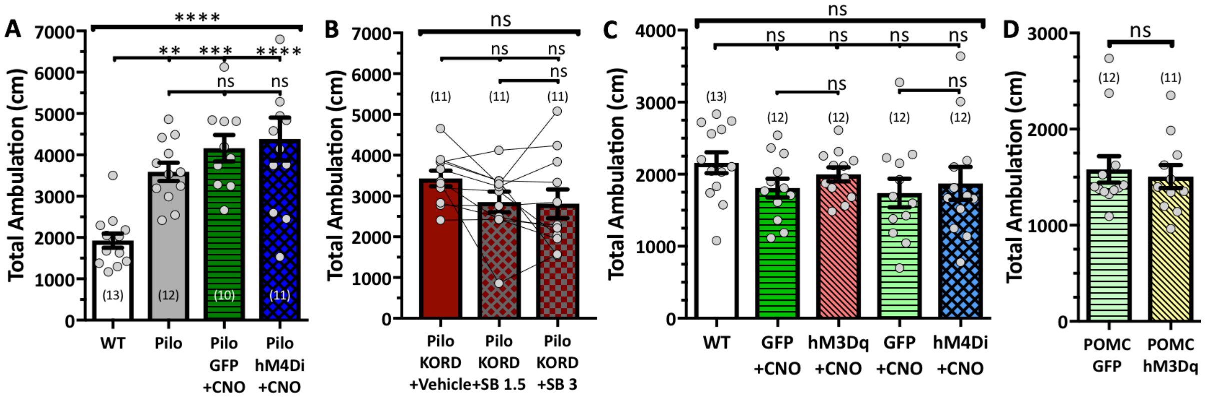

We analysed the total ambulation of the subjects in an open field paradigm to see if there were disease-, ligand-, or DREADD-related changes in locomotion. **A-B.** To assess ambulation, the SOR habituation trial was analysed for epileptic groups. Epileptic groups were hyperactive compared to WT mice; DREADDs and ligands did not affect the hyperactivity (**A:** $F_{(3,42)}$=11.83, p=<0.0001, one-way ANOVA with Tukey's multiple comparisons correction; **B:** $F_{(2,20)}$=3.438, p=0.052, repeating measures one-way ANOVA with Tukey's correction). **C-D.** To assess ambulation, control subjects were tested in a novel open field arena. **C.** Control groups expressing CamKIIα-DREADDs and their respective GFP control groups showed no significant changes in total ambulation compared to WT mice nor each other ($F_{(4,56)}$=1.03, p=0.400, one-way ANOVA with Tukey's correction). **D.** Total ambulation did not differ between the POMC-hM3Dq and -GFP groups (t_{21}=0.4055, p=0.689, Student's t-test). Data are represented as mean ± SEM. Each circle represents an animal (number of animals per group in parentheses). ns p>0.05, *p<0.05, **p<0.01, ***p<0.001, ****p<0.0001.

Supplementary Figure 9. SOR Object Interaction Metrics

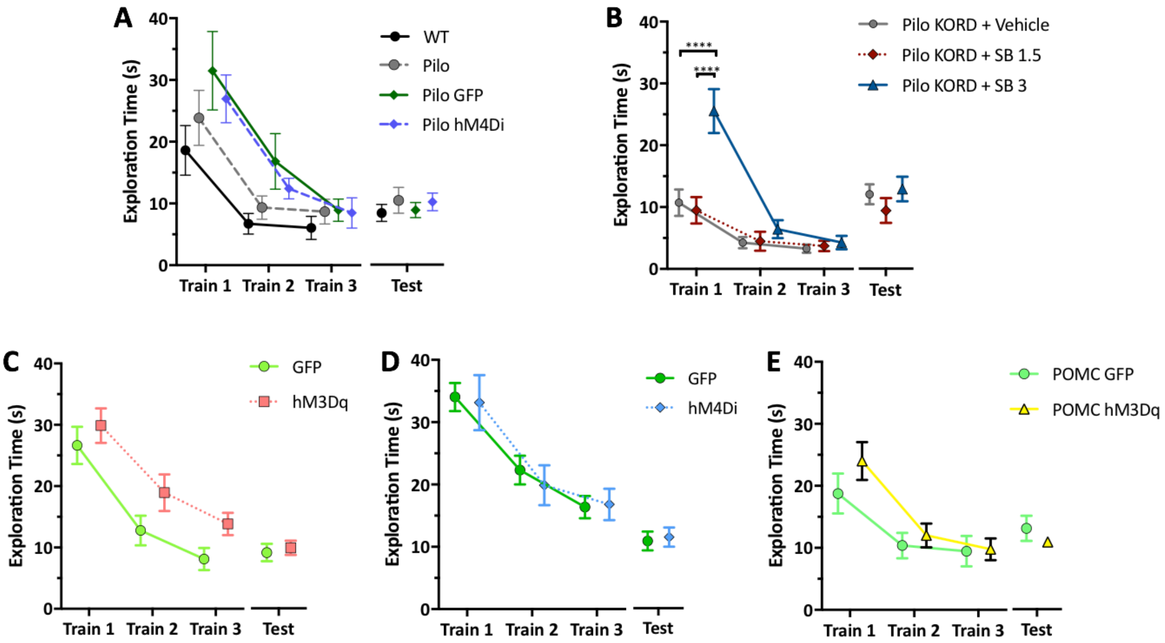

Total object exploration time during SOR training and testing trials. All groups demonstrated a steady decrease in exploration across their training, suggesting that the subjects were internalizing the environment appropriately during their training and not experiencing working memory deficits. Ordinary two-way ANOVAs for **A**, **C**, **D**, and **E** showed a significant effect of training (**A**: $F_{(2,92)}$=58.42, **C**: $F_{(2,44)}$=34.8, **D**: $F_{(2,44)}$=46.43, **E**: $F_{(2,44)}$=32.4, p<0.0001 for all) but no difference between groups (**A**: $F_{(3,46)}$=1.78, p=0.164; **C**: $F_{(1,22)}$=3.78, p=0.065; **D**: $F_{(1,22)}$=0.078, p=0.783; **E**: $F_{(1,69)}$=0.40, p=0.535), and no interaction (**A**: $F_{(6,92)}$=0.93, p=0.474; **C**: $F_{(2,44)}$=0.28, p=0.761; **D**: $F_{(2,44)}$=0.31, p=0.738; **E**: $F_{(2,44)}$=0.45, p=0.586). The two-way repeating measures ANOVA of training for **B** showed a significant effect of training ($F_{(2,20)}$=51.52, p<0.0001), treatment ($F_{(2,20)}$=6.40, p=0.007), and a significant interaction ($F_{(4,40)}$=9.677, p<0.0001) as the first training trial for the SB 3 group was significantly different from the vehicle and SB 1.5 groups (Tukey's multiple comparisons correction). This is likely because the 3 mg/kg treatment was the animals' first exposure to the box, while the SB 1.5 and vehicle treatments were later, counterbalanced exposures. Furthermore, there was no difference in the total time the subjects spent exploring the objects during the 3rd training trial, which was the trial that contributed to the discrimination index calculation (Tukey's multiple comparisons correction). The other trial that contributed to the DI was the test trial (right). There was no significant difference between the total exploration time in any group (**A**: $F_{(3,46)}$=0.403, p=0.752, one-way ANOVA; **B**: $F_{(2,20)}$=0.83, p=0.450, repeating measures one-way ANOVA; **C**: t_{22}=0.43, p=0.672, Student's t-test; **D**: t_{22}=0.29, p=0.772, Student's t-test, **E**: $t_{16.58}$=0.6862, p=0.502, Welch's t-test). Data are represented as mean ± SEM. ****p<0.0001

Supplementary Figure 10. EEG hM3Dq + CNO dose response curve

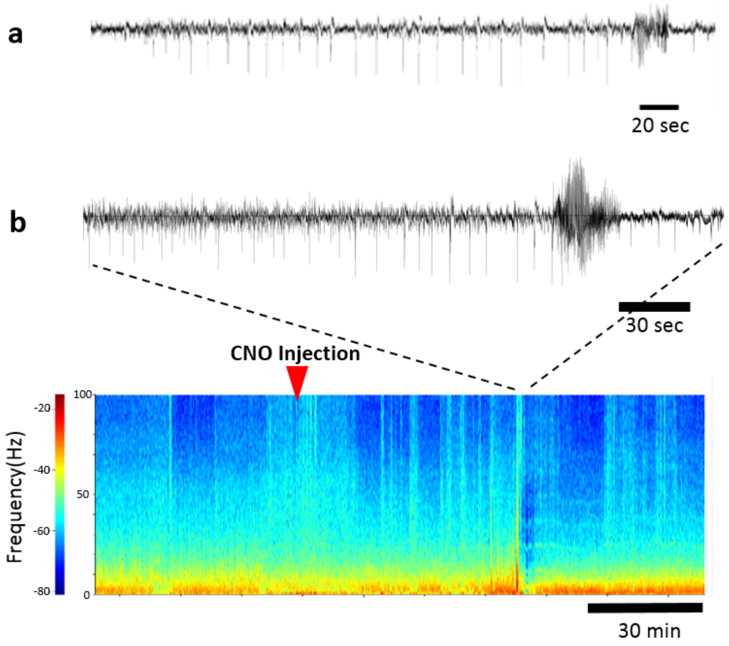

c	CNO Dose				
	0.3 mg/kg	1 mg/kg	3 mg/kg	10 mg/kg	30 mg/kg
# of Animals	0/6	0/6	0/6	3/6	4/6

To assess whether excitatory DREADDs might compromise behavioural performance by inducing seizures, control animals were injected in the dorsal DG bilaterally with the excitatory DREADD virus (CamKIIα-hM3Dq) and were implanted with EEG depth electrodes (see Supplementary Fig. 3 for implantation procedure). After 2 weeks of baseline recordings, mice received escalating doses of CNO (0.3, 1, 3, 10, and 30 mg/kg) that were administered (i.p.) with at least 1 day between injections to ensure no interaction between treatments. Custom written Matlab code was used to analyse the EEG traces, and events were confirmed with video monitoring. Spectral analysis was used to examine gamma activity over a 6-hour window immediately post-injection. No pathologic activity was detected in response to ≤ 3.0 mg/kg CNO. In response to elevated CNO doses (10 and 30 mg/kg), 4/6 of the subjects had EEG seizure discharges accompanied by Racine stage 2-5 behavioural seizures. **A**. EEG recordings during a stage 5 seizure event that occurred 4.5 h post-injection (10 mg/kg CNO). **B.** An EEG recorded event that was 1-hour post-injection (30 mg/kg CNO) with its corresponding spectrogram. **C.** Table showing the number of animals that had seizures at each CNO dose.
