# Supplementary material for: SYT1-associated neurodevelopmental disorder: a case series
Source: Brain. 2018 Aug 13;141(9):2576–91. doi: 10.1093/brain/awy209 (PMC6113648; doi:10.1093/brain/awy209)
Supplement: awy209_Supplementary_Materials [file awy209_supplementary_materials.zip › awy209-suppl_data/awy209_Suppl_data.pdf]

Supplementary Methods

Molecular Dynamics Simulation

MD simulations were carried out based on an NMR structure of the Synaptotagmin 1 C2B-domain including two bound Ca^{2+} atoms (PDB 1k5w) (Fernandez *et al.*, 2001) or mutant variants thereof (M302K, D303G, D365E, I367T and N370K; note that numbering used in figures and text follows human sequence for simplicity i.e. M303K, D304G, D366E, I368T and N371K) generated using MolSoft ICM Pro. All MD simulations were performed with the Gromacs molecular dynamics simulation package version 5.1.2 (Pronk *et al.*, 2013) using the Amber ff99SB-ILDN forcefield (Lindorff-Larsen *et al.*, 2010) under periodic boundary conditions and in a rhombic dodecahedron unit cell. Each system was solvated with simple point charge (SPC) water molecules (Berendsen, 1981) and heavy-hydrogen atoms (Feenstra *et al.*, 1999) were used throughout the equilibration and production MD stages to allow for the longer time step used in the production run. Na^{+} and Cl^{-} ions were added to neutralize the total charge of the systems at concentrations of 150 mM. The neighbour list, Coulomb and van der Waals interaction cut-offs were set to 1 nm and the particle mesh Ewald (PME) algorithm (Darden *et al.*, 1993) was utilised for long-range electrostatic interactions. All systems were first subjected to a 2000 step steepest descent energy minimization, alternately completing when the maximum force on any atom has reached 1000 kJ mol^{-1} nm^{-1}. A 1 ns NVT (constant Number of particles, Volume and Temperature) was then performed, heating the systems to 310 K using the Berendsen thermostat (Berendsen *et al.*, 1984) with position restraints on the protein. This was followed by a 5 ns NPT (constant Number of particles, Pressure and Temperature) equilibration run performed with restraints on the protein atoms using the V-rescale thermostat (Bussi *et al.*, 2007)and the Berendsen barostat (Berendsen *et al.*, 1984) A third equilibration phase with the V-rescale thermostat (Bussi *et al.*, 2007) (310 K) and the Parrinello-Rahman barostat (Parrinello and Rahman, 1981) was performed for 1 ns, using a 2 fs time step and without position restraints on any atom. The production runs were carried out under the same conditions as the final equilibration phase but using a 5 fs time step. The MD trajectories were simplified by extracting every 1000th ps and by removing all water molecules using the GROMACS trjconv utility. All simulations were carried out on the Victorian Life-science Super Computing Initiative (VLSCI) platform.

Materials for functional studies

SYT1-pHluorin (a pH-sensitive variant of GFP) was provided by Prof. V. Haucke (Leibniz Institute of Molecular Pharmacology, Berlin, Germany). QuikChange II Site-Directed Mutagenesis kit was from Agilent Technologies (Santa Clara, CA, USA). Neurobasal media, B-27 supplement, penicillin/streptomycin, Minimal Essential Medium (MEM), Lipofectamine 2000, goat anti-chicken IgY (H+L) Alexa Fluor 488, goat anti-chicken IgY (H+L) DyLight 550 and goat anti-rabbit IgG (H+L) Alexa Fluor 568 were obtained from ThermoFisher Scientific (Scoresby, Australia). Rabbit anti-SYT1 was from Synaptic Systems (Göttingen, Germany). Chicken anti-GFP was from AVES (Portland, OR, USA). 6-cyano-7-nitroquinoxaline-2,3-dione (CNQX) was from ENZO Life Sciences (Lausen, Switzerland). DL-2-Amino-5-phosphonopentanoic acid (AP5) was from Caymen Chemical (Ann Arbor, MI, USA). Bafilomycin A1 was from Toronto Research Chemicals (Toronto, Canada). All other reagents were obtained from Sigma-Aldrich (Castle Hill, Australia).

Primary hippocampal neuronal cultures

All procedures were approved by the Florey Animal Ethics Committee and performed in accordance with the guidelines of the National Health and Medical Research Council Code of Practice for the Care and Use of Animals for Experimental Purposes in Australia. Mouse colonies were maintained in a temperature controlled (≈ 21°C) room and group housed in individually ventilated cages on a 12 h light-dark cycle (lights on 0700–1900 h) with food and water available ad libitum. Animals were time mated overnight and visualisation of a vaginal plug on the following morning was considered as embryonic day (E) 0.5.

Dissociated primary hippocampal-enriched neuronal cultures were prepared from E16.5-18.5 C57BL/6J mouse embryos of both sexes by trituration of isolated hippocampi to obtain a single cell suspension, plated at a density of 3.5-5 $x10^5$ cells/coverslip on poly-D-lysine and laminin-coated 13 mm or 25 mm coverslips in 24 or 6 well plates respectively. Cultures were maintained in Neurobasal media supplemented with B-27, 0.5 mM L-glutamine and 1% v/v penicillin/streptomycin. After 72 hours, cultures were further supplemented with 1 µM cytosine β-d-arabinofuranoside to inhibit glial proliferation. Cells were transfected after 7-8 days in culture with Lipofectamine 2000 as described (Gordon *et al.*, 2011), with the following alterations: for 24 well plates, 1 µL Lipofectamine 2000 and 0.5 µg/DNA construct was used per well. Cells were utilised for fixation or live cell imaging assays after 13-16 days in culture.

Depolarisation, fixation and immunolabelling of neurons

For SYT1 expression and localisation assays, primary hippocampal neuronal cultures were first washed with saline imaging buffer (in mM: 136 NaCl, 2.5 KCl, 2 $CaCl_2$, 1.3 $MgCl_2$, 10 glucose, 10 HEPES, pH 7.4, supplemented with 10 μM CNQX and 50 μM AP5) and then either fixed immediately (basal), exposed to 50 mM KCl buffer (in mM: 88.5 NaCl, 50 KCl, 2 $CaCl_2$, 1.3 $MgCl_2$, 10 glucose, 10 HEPES, pH 7.4, supplemented with 10 μM CNQX and 50 μM AP5) for 30 seconds and then fixed immediately (KCl depolarisation), or exposed to 50 mM KCl buffer for 30 seconds and then allowed to recover in saline buffer for 2.5 minutes before being fixed (recover) (all performed at 37°C). Neurons were fixed on ice in 4% paraformaldehyde in phosphate-buffered saline (PBS) for 20 minutes, incubated at room temperature in 50 mM NH_4Cl in PBS for 10 minutes, washed with PBS and permeabilised with 0.1% v/v Triton-x100, 1% v/v bovine serum albumin (BSA) in PBS for 5 minutes. The cells were washed with PBS, blocked with 1% BSA in PBS for one hour before being incubated with antibodies in 1% BSA in PBS for 1-2 hours (with extensive washing with PBS after each incubation).

Fluorescence imaging of neurons

Fixed, immunolabelled cells or live neuronal cultures mounted in a Warner imaging chamber with embedded parallel platinum wires (RC-21BRFS) were placed on the stage of a Zeiss Axio Observer.Z1 epifluorescence microscope. All neurons were visualised using a Zeiss EC Plan-Neofluar 40x air objective (NA 0.75) or Zeiss Plan-Apochromat 63x oil-immersion objective (NA 1.4) with EGFP and DS Red filters at excitation wavelengths of 488 nm and 555 nm. Images were captured with a Zeiss Axiocam 506 mono camera and processed offline using Image J 1.51s software.

For live fluorescence imaging assays, we employed pHluorin (a pH-sensitive GFP) fused to the lumenal N-terminus of SYT1 variants, which reports exocytic rate when assayed in the presence of bafilomycin A1. pHluorin fluorescence is quenched inside the acidic lumen of synaptic vesicles but fluoresces upon exposure to the neutral extracellular fluid following fusion of vesicles with the plasma membrane during exocytosis. Fluorescence is quenched again following endocytosis as nascent synaptic vesicles are reacidified. This reacidification can be blocked by bafilomycin A1, which inhibits vATPase activity, causing the pHluorin to report all vesicles that have undergone fusion, which allows a measure of exocytic rate and total vesicle mobilisation.

Cultures were perfused with saline imaging buffer (as described above, supplemented with 1 μM bafilomycin A1) or high Ca^{2+} imaging buffer (as described above with 4 mM $CaCl_2$ in place of 2 mM $CaCl_2$, supplemented with 1 μM bafilomycin A1). Neurons were stimulated with a train of 1,200 action potentials (100 mA, 1ms pulse width) at 10 Hz to mobilise the entire recycling pool of vesicles before being challenged with alkaline imaging buffer (50 mM NH_4Cl substituted for 50 mM NaCl) to reveal total SYT1-pHluorin fluorescence. Images were captured at 4 s intervals.

To quantify synaptic SYT1 expression in immunolabelled neurons, identically sized regions of interest were placed over transfected SYT1 puncta and non-transfected puncta in the same field of view, along with background regions, and total fluorescence intensity measured. To determine somatic SYT1 expression levels, regions of interest were traced around somata of transfected and non-transfected cells, along with background regions, and average fluorescence intensity measured. The level of SYT1 overexpression was calculated by subtracting background autofluorescence and determining the ratio of transfected/non-transfected SYT1 expression levels. The diffuseness of fluorescence along axons was determined by calculating the coefficient of variation CV; as described in (Lyles *et al.*, 2006; Gordon and Cousin, 2013; Baker *et al.*, 2015), where n refers to the mean of 5 different >40 pixel axonal segments on a single field of view.

For time series images, regions of interest of identical size were placed over presynaptic boutons and the total fluorescence intensity within each region was monitored over time. Only regions that responded to action potential stimulation were selected for analysis. The pHluorin fluorescence change was calculated as $\Delta F/F_0$ and n refers to the number of individual coverslips examined.

Legend to patient videos

Video material provided by clinicians or parents, and reproduced online with additional parental consent. Clips have been selected to illustrate each patient's predominant movement abnormalities.

A) Patient 1, *SYT1* I368T

age 8 years:

Dystonia (four-limb, vocal), ballismus, action-induced chorea, repetitive leg kicking, stereotypies (hand-to-mouth), severe motor delay (cruising)

age 12 years:

Severe chorea, stereotypies (chest-beating), progress in motor abilities (walking short distances with broad-based gait)

B) Patient 2, *SYT1* I368T

age 4 years:

Dystonia (lower limb predominant), mild athetosis, stereotypies (hand-to-mouth, object mouthing, eye-poking)

C) Patient 3, *SYT1* D304G

age 21 years:

Stereotypies (repetitive tapping, chest-beating, object-mouthing), agitation

D) Patient 5, *SYT1* N371K

age 4 years:

Severe dystonia, facial grimacing, stereotypies (hand-to-mouth)

age 5 years:

Repetitive leg kicking, back-arching, action-induced involuntary movements, ballismus, stereotypies (hand biting)

E) Patient 7, *SYT1* N371K

age 2 years:

Dystonia, dyskinesia, stereotypies (back-arching, hand-to-mouth, tapping)

Switching from day-time happy and sociable disposition to night-time screaming episodes

Legend to molecular dynamics simulations movies.

1.3 µs simulations were performed on C2B models derived from the calcium-bound solution NMR structure (PDB 1k5w; note that amino acid numbering used in figure follows human sequence for simplicity) generated using Molsoft ICM Pro. .mp4 files of simulations of WT and mutant C2B domains.

A) $SYT1^{WT}$
B) $SYT1^{M303K}$
C) $SYT1^{D304G}$
D) $SYT1^{D366E}$
E) $SYT1^{I368T}$
F) $SYT1^{N371K}$

Supplementary Figure 1: <u>Molecular dynamics simulations trajectories.</u>

1.3 μs simulations were performed on C2B models derived from the calcium-bound solution NMR structure (PDB 1k5w; note that amino acid numbering used in figure follows human sequence for simplicity) generated using Molsoft ICM Pro. A) The root-mean-square deviations (RMSD) of the backbone atoms of each SYT1 variant C2B domain, compared to the starting structure (frame 0), was plotted over the complete trajectory of simulations. B, C) The Ca^{2+}-binding ability of the C2B domains was analysed by tracking the distances between the bound Ca^{2+} (calcium1, B; calcium 2, C) and the gamma carbon of Asp363 (equivalent to human Asp364) throughout the trajectories. Data displayed is the distance distributions of calcium 1 (B) (co-ordinated by Asp303, Asp309, Asp363 and Asp365 in 1k5w) and calcium 2 (C) (co-ordinated by Asp365, Asp363 and Asp371 in 1k5w) in the simulations of WT and mutant C2B domains.

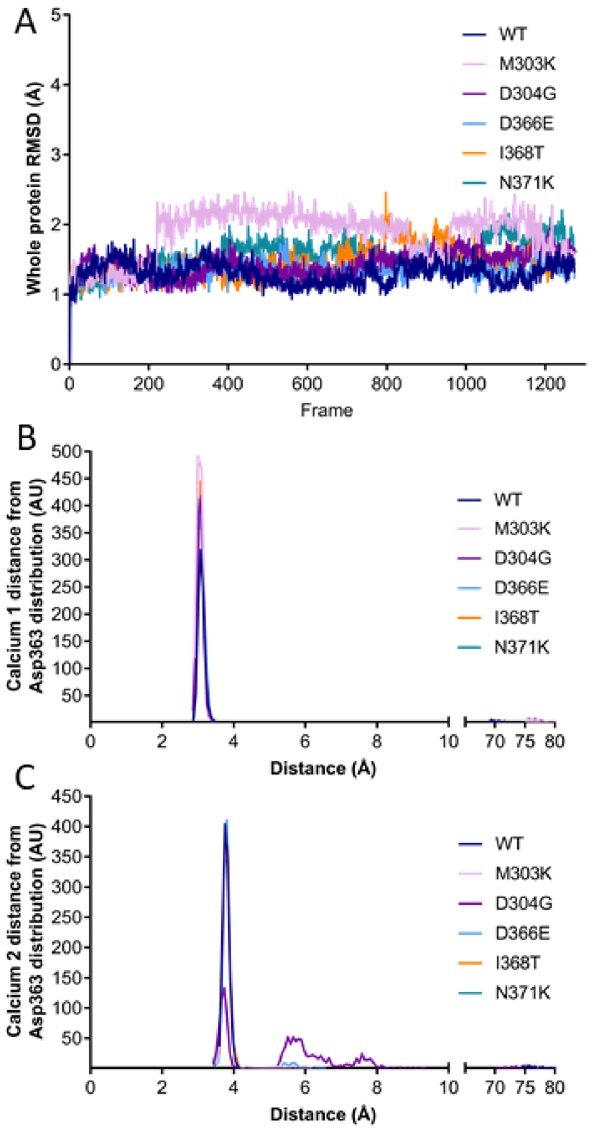

Supplementary Figure 2:

Facial photographs of patients with *de novo SYT1* mutations

Top row left to right: A. Patient three (early childhood and late adolescence), *SYT1* D304G; B. Patient four (age 2.5 years), *SYT1* D366E; C. Patient seven (age 3 years), *SYT1* N371K. Bottom row left to right (all *SYT1* I368T): D. Patient ten (age 2.5 years), E. Patient two (age 4 years); F. Patient eleven (age 6 years); G. (age 12 years). Consent to publish patient photographs has been obtained.

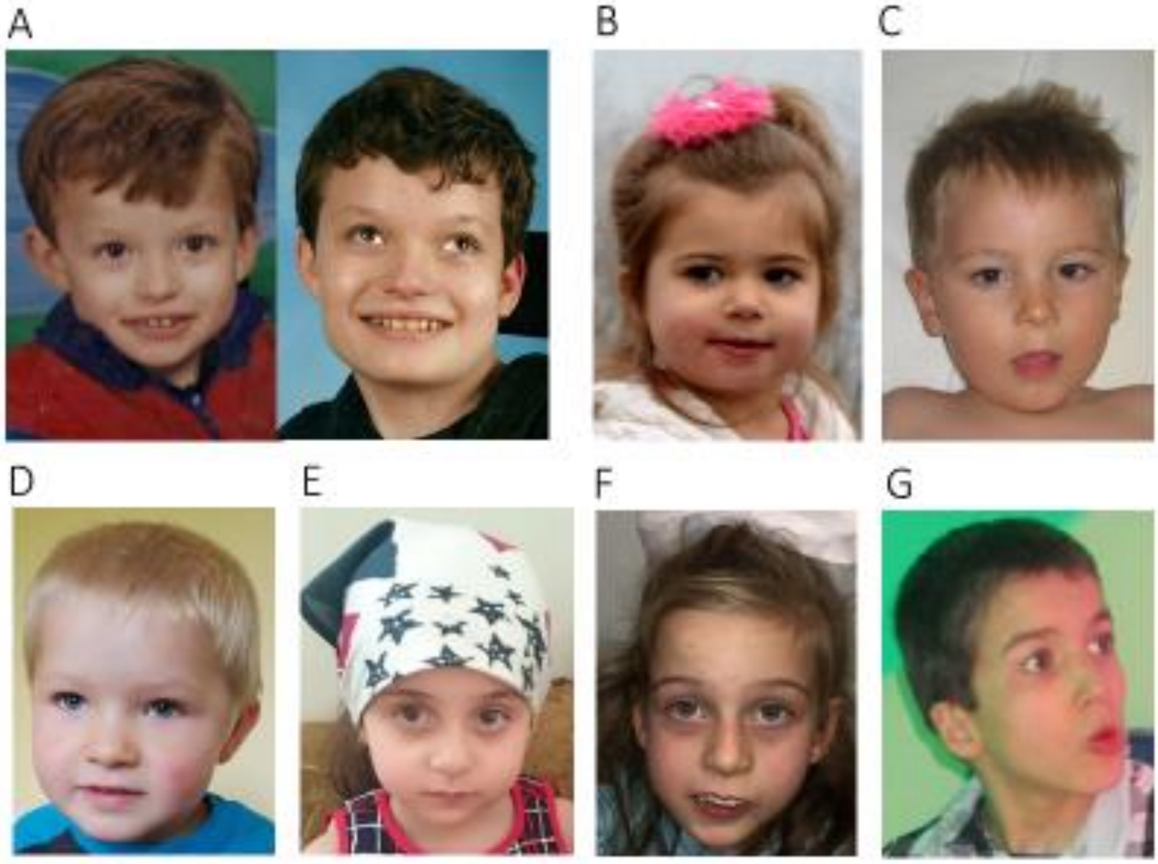

Supplementary Figure 3:

<u>MRI abnormalities of uncertain significance in one patient with *SYT1* mutation</u>

Clinical neuroimaging acquired for Patient Seven at age 2 years 1 month

A) Axial T1-weighted MRI

B) Axial FLAIR MRI

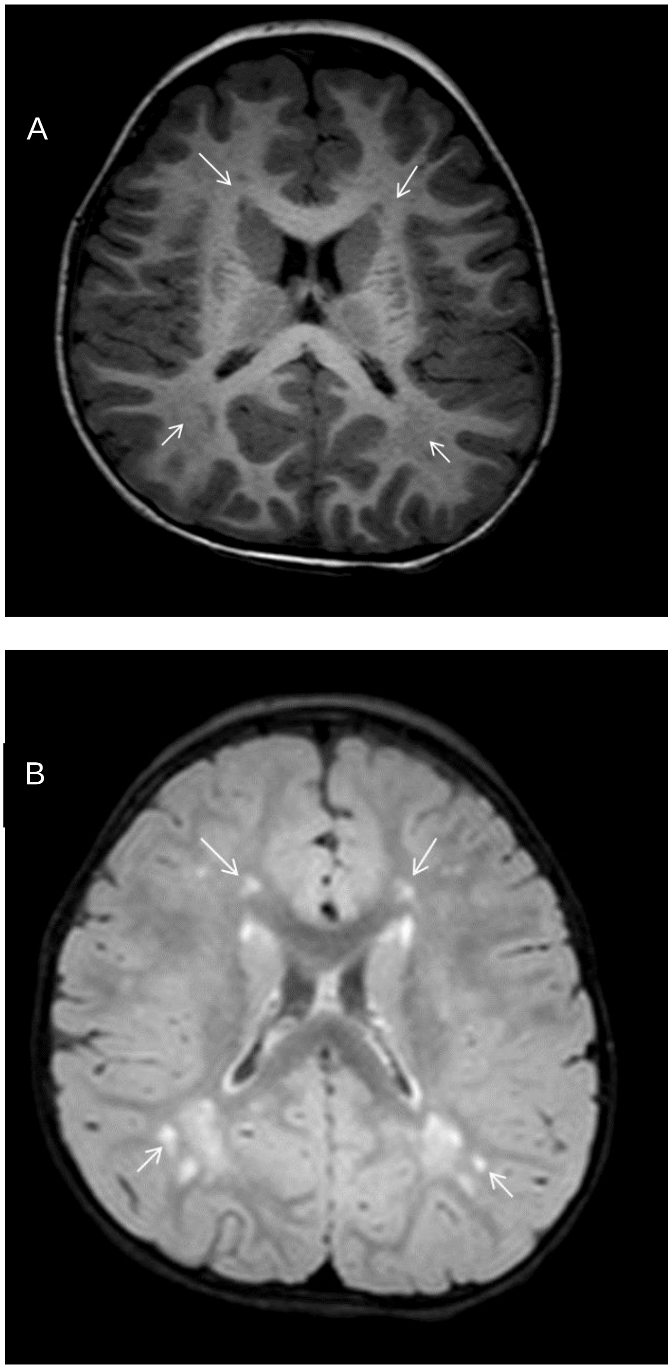

Supplementary Figure 4:

Somatic expression levels of SYT1 variants.

Cultured hippocampal neurons were transfected with SYT1-pHluorin variants, fixed at rest and immunolabelled for GFP and SYT1. Bar graph shows SYT1 immunofluorescence intensity in the soma of transfected neurons relative to non-transfected neurons in the same field of view. Data displayed as mean +/- SEM, n = 5-8. *p < 0.05 compared to WT, one-way ANOVA with Dunnett's multiple comparison test.

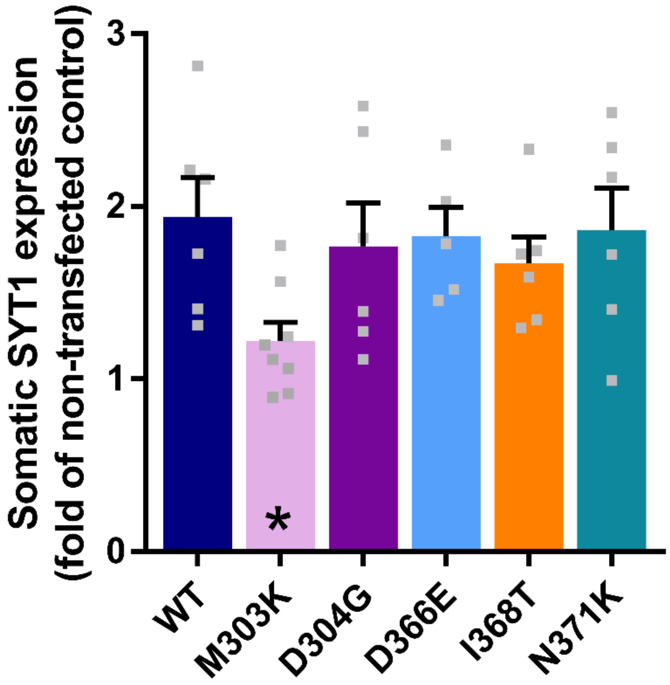

Supplementary Table 2:

Phenotypic features reported for at least one case of *SYT1* mutation

**System**	**HPO term**	**Feature**
Eye		
	HP:0000565	Esotropia
	HP:0000540	Hypermetropia
	HP:0000486	Strabismus
	HP:0000639	Nystagmus
Cutaneous		
	HP:0025247	Dermoid cyst
Cardiovascular		
	HP:0001631	Atrial septal defect
Respiratory		
	HP:0010536	Sleep apnea
	HP:0001601	Laryngomalacia
	HP:0002883	Hyperventilation
Musculoskeletal		
	HP:0002650	Scoliosis
	HP:0008081	Pes valgus
	HP:0002938	Lumbar hyperlordosis
	HP:0001883	Talipes
	HP:0008081	Pes valgus
Gastrointestinal		
	HP:0002020	Gastroesophageal reflux
	HP:0012450	Chronic constipation
	HP:0011968	Feeding difficulties
Neurological		
	HP:0000733	Stereotypy
	HP:0012169	Self-biting
	HP:0000742	Self-mutilation
	HP:0100716	Self-injurious behavior
	HP:0012168	Head-banging

	HP:0100710	Impulsivity
	HP:0002353	EEG abnormality
	HP:0002451	Limb dystonia
	HP:0007098	Paroxysmal choreoathetosis
	HP:0001344	Absent speech
	HP:0002465	Poor speech
	HP:0001263	Global developmental delay
	HP:0002457	Abnormal head movements
	HP:0100022	Abnormality of movement
	HP:0002828	Multiple joint contractures
	HP:0100021	Cerebral palsy
	HP:0002072	Chorea
Connective Tissue		
	HP:0002828	Multiple joint contractures

Supplementary Table 2:

Primers used for site-directed mutagenesis of SYT1-pHluorin.

Rat syt1 Ref Seq: NC_005106.4; UniProtKB - P21707 (SYT1_RAT)

Mutated bases are in bold and underlined. Mutations refer to human sequence.

Mutation	Primer	Sequence
M303K	Forward	CCAAGAACCTGAAGAAGA**A**GGATGTGGGTGGC
	Reverse	GCCACCCACATCC**T**TCTTCTTCAGGTTCTTGG
D304G	Forward	GAACCTGAAGAAGATGG**G**TGTGGGTGGCTTATCTG
	Reverse	CAGATAAGCCACCCACA**C**CCATCTTCTTCAGGTTC
D366E	Forward	CTGTTTTGGACTATGA**G**AAGATTGGCAAGAACGACGCG
	Reverse	CGCGTCGTTCTTGCCAATCTT**C**TCATAGTCCAAAACAG
N371K	Forward	CAAGATTGGCAAGAA**G**GACGCGATCGGC
	Reverse	GCCGATCGCGTC**C**TTCTTGCCAATCTTG

Supplementary references

Baker K, Gordon SL, Grozeva D, van Kogelenberg M, Roberts NY, Pike M, *et al.* Identification of a human synaptotagmin-1 mutation that perturbs synaptic vesicle cycling. J Clin Invest 2015; 125(4): 1670-8.

Berendsen HJC, Postma JPM, Vangunsteren WF, Dinola A, Haak JR. Molecular-Dynamics with Coupling to an External Bath. J Chem Phys 1984; 81(8): 3684-90.

Berendsen HJC, Postma, J. P. M., van Gunsteren, W. F., Hermans, J. . Interaction Models for Water in Relation to Protein Hydration. In: Pullman B, editor. Intermolecular Forces: Proceedings of the Fourteenth Jerusalem Symposium on Quantum Chemistry and Biochemistry 1981; Jerusalem, Israel: Springer Netherlands: Dordrecht; 1981. p. 331-42.

Bussi G, Donadio D, Parrinello M. Canonical sampling through velocity rescaling. J Chem Phys 2007; 126(1).

Darden T, York D, Pedersen L. Particle Mesh Ewald - an N.Log(N) Method for Ewald Sums in Large Systems. J Chem Phys 1993; 98(12): 10089-92.

Feenstra KA, Hess B, Berendsen HJC. Improving efficiency of large time-scale molecular dynamics simulations of hydrogen-rich systems. J Comput Chem 1999; 20(8): 786-98.

Fernandez I, Arac D, Ubach J, Gerber SH, Shin O, Gao Y, *et al.* Three-dimensional structure of the synaptotagmin 1 C2B-domain: synaptotagmin 1 as a phospholipid binding machine. Neuron 2001; 32(6): 1057-69.

Gordon SL, Cousin MA. X-linked intellectual disability-associated mutations in synaptophysin disrupt synaptobrevin II retrieval. J Neurosci 2013; 33(34): 13695-700.

Gordon SL, Leube RE, Cousin MA. Synaptophysin is required for synaptobrevin retrieval during synaptic vesicle endocytosis. J Neurosci 2011; 31(39): 14032-6.

Lindorff-Larsen K, Piana S, Palmo K, Maragakis P, Klepeis JL, Dror RO, *et al.* Improved side-chain torsion potentials for the Amber ff99SB protein force field. Proteins 2010; 78(8): 1950-8.

Lyles V, Zhao Y, Martin KC. Synapse formation and mRNA localization in cultured Aplysia neurons. Neuron 2006; 49(3): 349-56.

Parrinello M, Rahman A. Polymorphic Transitions in Single-Crystals - a New Molecular-Dynamics Method. J Appl Phys 1981; 52(12): 7182-90.

Pronk S, Pall S, Schulz R, Larsson P, Bjelkmar P, Apostolov R, *et al.* GROMACS 4.5: a high-throughput and highly parallel open source molecular simulation toolkit. Bioinformatics 2013; 29(7): 845-54.
